# Exon junction complex (EJC) core genes play multiple developmental roles in *Physalis floridana*

**DOI:** 10.1007/s11103-018-0795-9

**Published:** 2018-11-13

**Authors:** Pichang Gong, Jing Li, Chaoying He

**Affiliations:** 1 State Key Laboratory of Systematic and Evolutionary Botany, Institute of Botany, Chinese Academy of Sciences, Beijing, 100093 China; 2 University of Chinese Academy of Sciences, Beijing, 100049 China

**Keywords:** Pre-mRNA splicing, Defense-related process, Exon junction complex (EJC), Functional evolution, Pollen maturation, *Physalis floridana*

## Abstract

**Key message:**

Molecular and functional characterization of four gene families of the *Physalis* exon junction complex (EJC) core improved our understanding of the evolution and function of EJC core genes in plants.

**Abstract:**

The exon junction complex (EJC) plays significant roles in posttranscriptional regulation of genes in eukaryotes. However, its developmental roles in plants are poorly known. We characterized four EJC core genes from *Physalis floridana* that were named *PFMAGO, PFY14, PFeIF4AIII* and *PFBTZ*. They shared a similar phylogenetic topology and were expressed in all examined organs. PFMAGO, PFY14 and PFeIF4AIII were localized in both the nucleus and cytoplasm while PFBTZ was mainly localized in the cytoplasm. No protein homodimerization was observed, but they could form heterodimers excluding the PFY14-PFBTZ heterodimerization. Virus-induced gene silencing (VIGS) of *PFMAGO* or *PFY14* aborted pollen development and resulted in low plant survival due to a leaf-blight-like phenotype in the shoot apex. Carpel functionality was also impaired in the *PFY14* knockdowns, whereas pollen maturation was uniquely affected in *PFBTZ*-VIGS plants. Once *PFeIF4AIII* was strongly downregulated, plant survival was reduced via a decomposing root collar after flowering and Chinese lantern morphology was distorted. The expression of *Physalis* orthologous genes in the *DYT1*-*TDF1*-*AMS*-*bHLH91* regulatory cascade that is associated with pollen maturation was significantly downregulated in *PFMAGO*-, *PFY14*- and *PFBTZ*-VIGS flowers. Intron-retention in the transcripts of *P. floridana dysfunctional tapetum1* (*PFDYT1*) occurred in these mutated flowers. Additionally, the expression level of *WRKY* genes in defense-related pathways in the shoot apex of *PFMAGO-* or *PFY14-*VIGS plants and in the root collar of *PFeIF4AIII*-VIGS plants was significantly downregulated. Taken together, the *Physalis* EJC core genes play multiple roles including a conserved role in male fertility and newly discovered roles in Chinese lantern development, carpel functionality and defense-related processes. These data increase our understanding of the evolution and functions of EJC core genes in plants.

**Electronic supplementary material:**

The online version of this article (10.1007/s11103-018-0795-9) contains supplementary material, which is available to authorized users.

## Introduction

The exon junction complex (EJC) is a vital surveillance system in posttranscriptional regulation of mRNAs in eukaryotes, including pre-mRNA splicing, matured mRNA transport, non-sense mediated mRNA decay (NMD) and protein translation enhancement (Tang et al. 2004; Moore 2005; Ashton-Beaucage et al. 2010; Roignant and Treisman 2010; Ghosh et al. 2012; Kervestin and Jacobson 2012; Chazal et al. 2013; Le Hir et al. 2016). EJC is a multiple protein complex including both core and peripheral factors, which tightly bind to mRNAs in a splicing-dependent manner, and this protein complex establishes a molecular link between splicing and cytoplasmic processes (Le Hir et al. 2016). The EJC core consists of four proteins, including MAGOH NASHI (MAGO), RNA-binding motif 8A (RBM8A, also known as Y14), eukaryotic initiation factor 4A3 (eIF4AIII or eIF4A3) and metastatic lymph node 51 (MLN51/CASC3) in mammals or Barentsz (BTZ) in *Drosophila* (Ballut et al. 2005; Tange et al. 2005). These four proteins form intertwined interaction networks by stably clamping mRNA molecules in a sequence-independent manner during the posttranscriptional regulation process (Andersen et al. 2006; Bono et al. 2006). They provide a basic platform for peripheral factors to combine once the nuclear splicing machinery is formed (Le Hir et al. 2016).

Knowledge of EJC core proteins comes mainly from animal studies performed during the last 20 years. Both MAGO and Y14 are evolutionarily conserved proteins (Hachet and Ephrussi 2001; Mohr et al. 2001). Crystal structures of MAGO-Y14 heterodimer in *Drosophila* and humans show that the two proteins mainly associate with spliced mRNA (Lau et al. 2003; Shi and Xu 2003). *MAGO* is one of the strict maternal effect genes that plays a significant role in germ cell formation by regulating *oskar* mRNA localization in *Drosophila* (Boswell et al. 1991; Newmark and Boswell 1994; Micklem et al. 1997; Newmark et al. 1997). This protein also plays important roles in other developmental processes. It participates in hermaphrodite germ-line sex determination in *Caenorhabditis elegans* (Li et al. 2000) and controls cyclin-dependent kinase (Cdks) activity and proliferation and expansion of neural crest-derived melanocytes in mice (Inaki et al. 2011; Silver et al. 2013). It is also involved in the hedgehog signaling pathway in *Drosophila* (Garcia–Garcia et al. 2017). Y14/RBM8A, as the obligate interacting partner of the MAGO protein, exercises similar functions in *oskar* mRNA localization or sex-determination (Mohr et al. 2001; Fribourg et al. 2003; Kawano et al. 2004; Parma et al. 2007; Lewandowski et al. 2010). It targets neuronal genes to regulate anxiety behaviors in mice (Alachkar et al. 2013). Y14 provides a regulatory link between pre-mRNA splicing and snRNP biogenesis by modulating methylosome activity (Chuang et al. 2011). It inhibits the mRNA-decapping activity by interacting with decapping factors (Chuang et al. 2013, 2016) and modulates DNA damage sensitivity in an EJC-independent manner (Lu et al. 2017). The eIF4AIII belongs to the DEAD-box RNA helicase family and contributes almost entirely to the interface with RNA by forming an ATP­dependent RNA clamp with its two conserved domains (Chan et al. 2004; Andersen et al. 2006; Bono et al. 2006; Gehring et al. 2009). The eIF4AIII interacts not only with the MAGO-Y14 heterodimer but also with BTZ, providing a molecular link among EJC core components (Palacios et al. 2004). Similar to MAGO and Y14 proteins, eIF4AIII is also required for *oskar* mRNA localization to the posterior end of mammalian oocytes (Palacios et al. 2004). Transcriptome­wide CLIP­seq (crosslinking-immunoprecipitation and high-throughput sequencing) in Hela cells revealed that the purine-rich sequences motif GAAGA is the potential binding site of eIF4AIII (Saulière et al. 2012). Furthermore, eIF4AIII was found to be a specific translation initiation factor for CBC (cap-binding complex)-dependent translation (Choe et al. 2014). MLN51, the ortholog of BTZ, is a breast cancer protein and overexpressed in breast carcinomas (Tomasetto et al. 1995), and BTZ in *Drosophila* is also vitally responsible for *oskar* mRNA localization (van Eeden et al. 2001). However, many animal BTZ orthologs are mainly involved in promoting protein translation (Degot et al. 2004; Ha et al. 2008; Chazal et al. 2013). All the pre-EJC components can bind the nascent transcripts of intron-containing or intronless genes in *Drosophila* (Choudhury et al. 2016), and these four core proteins are assembled as a complex, exerting certain posttranscriptional roles as a function, for example, to affect gene splicing. This assumption is further supported by observations that the mutation or knockdown of each gene in *D. melanogaster* results in the skipping of several exons of *mapk* pre-mRNA (Ashton-Beaucage et al. 2010; Roignant and Treisman 2010). Knockdown of either EJC core component genes causes widespread and similar alternative splicing changes in mammalian cells (Wang et al. 2014).

Compared to animals, orthologs of the EJC core in plants have been largely overlooked since the first plant *MAGO* gene was isolated in rice (Swidzinski et al. 2001). To begin with, limited evidence suggests several essential roles of EJC genes in plants. *MAGO, Y14* and *eIF4AIII* genes are involved in the growth, development and reproduction processes in some plant species (Park et al. 2009; Boothby and Wolniak 2011; Gong and He 2014; Gong et al. 2014a; Ihsan et al. 2015; Huang et al. 2016). *MAGO* and *Y14* are regulated by ethylene and jasmonate and *MAGO* may be involved in the aggregation of rubber particles in *Hevea brasiliensis* (Yang et al. 2016). *Arabidopsis* eIF4AIII is co-localized with AtMAGO and AtY14 proteins, and its subcellular localization mode is altered in response to hypoxia or by different phosphorylation states (Koroleva et al. 2009; Cui et al. 2016). In rice, *MAGO, Y14* and *eIF4AIII* are involved in the splicing of *UNDEVELOPED TAPETUM1* (*OsUDT1*) transcripts (Gong and He 2014; Huang et al. 2016), a key transcription factor in anther and pollen development (Jung et al. 2005). There are no reports on the plant *BTZ* gene as yet. Moreover, the molecular and functional evolution of plant EJC genes is poorly known, although the MAGO and Y14 family have undergone slow coevolution to maintain their obligate heterodimerization (Gong et al. 2014b), and functional evolution of the duplicated MAGO-Y14, especially in *Oryza*, is associated with adaptive evolution (Gong and He 2014). Therefore, EJC core genes in plants require further study to understand the functional evolution of gene expression components in eukaryotes.

The fruit of *Physalis* spp. (Solanaceae) has a lantern structure. It is produced via heterotopic expression of MADS-box gene 2 in *Physalis floridana* (*MPF2*), and this gene is also associated with pollen development (He and Saedler 2005). MPF2 interacts with *P. floridana* MAGO (PFMAGO) proteins, and this finding helps to explain the role of MPF2 in male fertility (He et al. 2007), thus providing a new insight into the understanding of the origin of the Chinese lantern. To understand the developmental roles of plant EJC core genes, as well as potential roles in the development of the Chinese lantern, in this study, *P. floridana* EJC core genes (*PFMAGO, PFY14, PFeIF4AIII* and *PFBTZ*) were characterized in multiple ways, including phylogeny, gene expression, subcellular localization, protein–protein interaction (PPI) and developmental roles. We found that these genes shared similar evolutionary patterns and expression modes, but they also had many differences. Gene-specific downregulation demonstrated that these genes could affect multiple developmental processes, including a conserved role in male fertility determination and new functions in female fertility, Chinese lantern development and defense-related pathways. In particular, severe downregulation of *PFMAGO, PFY14* and *PFeIF4AIII* produced lethal phenotypes. Accordingly, the transcripts of some genes in the related pathways were found to be modified either at expression levels or transcript forms, indicating the conserved roles of EJC core in mRNA metabolism, such as stability and splicing of transcripts.

## Materials and methods

### Plant materials

*Physalis floridana* P106 (He and Saedler 2005) was grown in a greenhouse at the State Key Laboratory of Systematic and Evolutionary Botany of the Chinese Academy of Sciences (Beijing, China) under long days (16 h light and 8 h dark) at a constant temperature of 23 °C. *Nicotiana benthamiana* plants were grown in an incubator (RXZ-380C; Ningbo) under long days (16 h light and 8 h dark) at a temperature of 25 °C and 22 °C.

### RNA extraction

Roots and leaves were obtained from 3-month-old seedlings for organ-specific gene expression assays. Other organs/tissues were harvested as indicated, and immediately frozen and stored in liquid nitrogen. Total RNA was extracted using the SV Total RNA Isolation System (Promega, USA).

### Sequence isolation

About 2.0 µg RNA from floral buds was treated with a RNase-free DNase I Kit (Promega, USA) in a 10 µl volume to remove genomic DNA contamination. The first-strand cDNA was synthesized with the oligo (dT)_18_ primers using M-MLV cDNA synthesis kit (Invitrogen, China) following product instructions in a 20 µl volume. Full-length cDNA sequences of the involved genes were obtained by a routine RT-PCR method using gene-specific primers (Supplementary Table S1). Each amplified fragment was purified using the High Pure PCR Product Purification Kit (Roche, Switzerland). Purified fragments were ligated into the *pEASY®-*Blunt Cloning vector (TransGen Biotech, China) and transformed into *Trans10* chemically competent cells (TransGen Biotech, China). Sequencing was performed by Taihe Biotech, China.

### Multiple sequence alignment (MSA) and phylogenetic analysis

MSA of the involved gene families was performed by BioEdit software version 5.09 (Hall 1999). Neighbor-joining (NJ) phylogenetic tree was constructed by MEGA 6.0 software (Tamura et al. 2013) with parameters of maximum composite likelihood model, pairwise deletion and bootstrap values for 1000 replicates.

### Gene expression analyses

The first strand cDNA was synthesized from total RNAs of the indicated biological samples. For semi-quantitative RT-PCR, a 1.0 µl aliquot of the synthesized cDNA stock solution was used. Electrophoresis of PCR products was run on 1.0% agarose gels, photographed using an ultraviolet imager, and the typical results were presented. Quantitative RT-PCR (qRT-PCR) assay was carried out on an Mx3000p Real-time RT-PCR instrument (Stratagene, Germany) using SYBR® *Premix Ex Taq*™ (TAKARA, Japan) at an amplification procedure consisting of 95 °C for 30 s, followed by 46 cycles of 95 °C for 5 s, 56 °C for 20 s, and 72 °C for 20 s, which were followed by a dissociation curve analysis. The *PFACTIN* gene was used as the internal reference in the RT-PCR analyses.

### Yeast two-hybrid assays

ORFs of *PFMAGO1, PFMAGO2, PFY14, PFeIF4AIII*, and *PFBTZ* were respectively inserted into the *pGADT7* or *pGBKT7* vectors to form the prey or bait constructs, and then small yeast transformation mediated by LiAC was performed using manufacturer instructions (Clontech, USA). To confirm the protein interactions, nonlethal β-galactosidase activity analysis was performed on strict defective medium SD/-Leu-Trp-His-Ade as described previously (He et al. 2007).

### Transient expression assays

For the subcellular localization, the ORF of *PFMAGO1, PFMAGO2, PFY14*, and *PFeIF4AIII* was inserted into the plant binary vector *pCAMBIA1302* with restriction sites *Nco*I/*Spe*I, and fused to the N-terminal of GFP to form fusion proteins. *PFBTZ* coding sequence was inserted into another binary vector *pSuper1300* with *Xba* I/ *Kpn* I to form the PFBTZ-GFP fusion protein due to limitation of restriction sites. Each fusion protein vector was agroinfiltrated into epidermal cells of *Nicotiana benthamiana*. A construct that produced only GFP was used as the control.

For the bimolecular fluorescence complementation (BiFC) assay, ORFs of *MPF2, PFMAGO1, PFMAGO2, PFY14, PFeIF4AIII* and *PFBTZ* were cloned into the *pSPYNE-35S* or *pSPYCE-35S* plant binary vector pair using *Xba* I/*Kpn* I to form either the N- or C-terminal ends of YPF protein. Combination of the N- and C-terminal resultant vectors was cotransformed into leaf epidermal cells of *Nicotiana benthamiana* by agroinfiltration (Walter et al. 2004). Fluorescence signals of GFP or YFP were observed by a confocal laser scanning microscope (Olympus FV1000 MPE, Japan).

### VIGS analysis

VIGS procedures were performed as previously described (Zhang et al. 2014). The trigger sequence for each gene silencing (Supplementary Fig. S1) was cloned into the *TRV2*. Leaves in 14DAI were used to identify gene silencing *P. floridana* lines by qRT-PCR. The gene expression in flowers, shoot apex, or root collar of each gene-specific VIGS plant was investigated using primers in Supplementary Table S1. The expression of target genes from 16 to 80 flowers or at least three independent samples of shoot apex or root collar was checked to evaluate the extent of gene silencing.

### Morphological assays

Plant morphology was photographed using a digital single lens reflex camera (D7000, Nikon, Japan). The leaf primordium, root collar, flower, Chinese lantern, fruits and pollen grains were photographed using a Zeiss microscope (Zeiss, Germany). Pollen grain maturation was investigated using iodine–potassium iodide (I_2_–KI) staining.

### Statistical analyses

Each experiment/measurement was performed using at least three independent biological replicates unless stated otherwise. Mean and standard deviation were presented. A Student’s two-tailed *t*-test was used for statistical analysis, which was performed using IBM SPSS Statistics for Windows, Version 24.0 (IBM Corp, New York, USA).

## Results

### Isolation and sequencing analyses of *P. floridana* EJC core cDNAs

*MAGO* homologous genes were previously isolated in *P. floridana* and named *PFMAGO1* (EF205415) and *PFMAGO2* (EF205416). These two paralogs shared high sequence identity (He et al. 2007). In this study, we rescreened a *P. floridana* transcriptome using these two *PFMAGOs* as the template and no additional homologs were found. This suggests that only two *MAGO*-like genes exist in the *P. floridana* genome. For the other three EJC core genes, we used the *Oryza sativa RBM8A*/*Y14* (KF051016/KF051017), *Arabidopsis thaliana AteIF4AIII* (NM_104029) and *Homo sapiens MLN51*/*CASC3* (XM_005257163) to blast the *P. floridana* transcriptome. Only one copy of each gene was hit and these, respectively, were named *PFY14, PFeIF4AIII* and *PFBTZ* (Supplementary Table S2). The full-length cDNA of these genes was then isolated using RT-PCR, revealing that the open reading frame (ORF) of *PFY14, PFeIF4AIII* and *PFBTZ* encoded 188-, 391-, and 688-amino acid (aa) peptides, respectively (Supplementary Table S2). The predicted average hydropathicity values of the four EJC core homologous proteins were all negative (Supplementary Table S2), indicating that they were hydrophilic with a potential capability of shuttling between the nucleus and the cytoplasm.

Multiple sequence alignment (MSA) of each protein family from 17 species was displayed including four animals and 13 plants (Supplementary Table S3 and Supplementary Fig. S2–S5). MAGO proteins were highly conserved (from 9 to 151 aa), while they did not contain any defined motifs. However, four conserved leucine residues that constituted a potential leucine zipper in the C-terminus were found (Pozzoli et al. 2004; Chu et al. 2009; Supplementary Fig. S2). Y14 proteins had a central RNA recognition motif (RRM, amino acids 77–170) flanked by highly divergent N- and C-terminal regions, and the most conserved regions in the RRM were RNP1 and RNP2 motifs (Lau et al. 2003; Shi and Xu 2003; Chu et al. 2009; Supplementary Fig. S3). The eIF4AIII protein belonged to DEAD-box helicase family which was characterized by the conserved motif Asp-Glu-Ala-Asp (DEAD). MSA showed that this protein family contained two RecA-like domains joined by an 11-residue linker (LVKRDELTLEG) and they possessed nine highly conserved motifs along with an N-terminal flanking sequence (Andersen et al. 2006; Cordin et al. 2006; Huang et al. 2016; Supplementary Fig. S4). The eIF4AIII in dicots had a conserved DESD motif instead of the DEAD motif occurring in animals, algae and monocots (Supplementary Fig. S4). The conserved region of the BTZ family was about 80-aa long and named SELOR or the eIF4AIII binding domain (Degot et al. 2004; Supplementary Fig. S5).

The conserved region size of each protein was summarized and their tertiary structures were predicted (Supplementary Fig. S6). PFBTZ was not predicted to have any secondary structure elements, whereas the other three EJC core proteins were composed of a variable number of α-helices and β-strands (Supplementary Fig. S6a). In the simulated *Physalis* EJC core tetrameric structure based on human EJC structure, the PFMAGO-PFY14 heterodimer kept the two domains of PFeIF4AIII in a closed conformation and the conserved SELOR domain of PFBTZ was wrapped around two domains of PFeIF4AIII (Supplementary Fig. S6b). This is similar to the structure of mammalian EJC, suggesting conserved roles of mRNA metabolism in the *Physalis* EJC core.

### Evolutionary analysis of the EJC core protein families

**Fig. 1:**
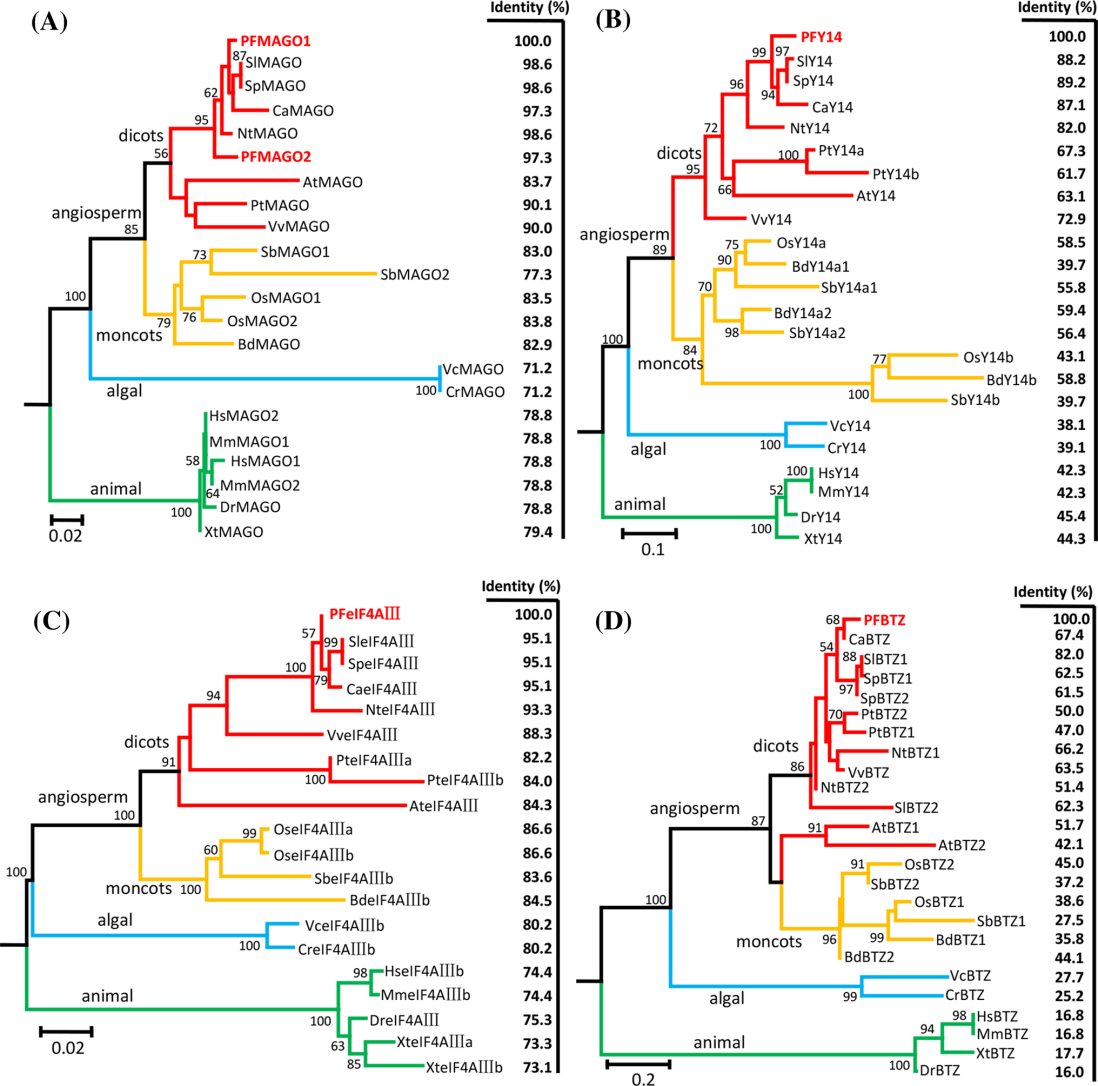
Phylogenetic reconstruction of EJC core genes. **a** Neighbor-Joining (NJ) tree of MAGO protein family. **b** NJ tree of the Y14 protein family. **c** NJ tree of the eIF4AIII protein family. **d** NJ tree of the BTZ protein family. The sequence identity compared with *Physalis* orthologs was indicated on the right panel of each tree. The amino acid sequences include the orthologs from *Physalis floridana* (PF), *Solanum lycopersicon* (Sl), *Solanum pennellii* (Sp), *Capsicum annuum* (Ca), *Nicotiana tomentosiformis* (Nt), *Vitis vinifera* (Vv), *Populus trichocarpa* (Pt), *Arabidopsis thaliana* (At), *Oryza sativa* (Os), *Sorghum bicolor* (Sb), *Brachypodium distachyon* (Bd), *Volvox carteri nagariensis* (Vc), *Chlamydomonas reinhardtii* (Cr), *Homo sapiens* (Hs), *Mus musculus* (Mm), *Danio rerio* (Dr), and *Xenopus tropicalis* (Xt). Information on these genes is presented in Supplementary Table S3

To reveal the evolutionary relationships of *Physalis* EJC core genes, sequence identity and phylogenetic analyses were performed based on the MSAs of these four protein families (Supplementary Fig. S2–S5). The sequence identity of each *Physalis* gene with other homologs was reduced within its own gene family, which was dependent on the evolutionary distance (Fig. 1). MAGO and eF4AIII families were relatively conserved since their sequence identity ranged from 73.1 to 98.6% (Fig. 1a, c), while the Y14 (38.1–89.2%) and BTZ (16.0–82.0%) families were highly divergent (Fig. 1b, d). The BTZ family was the most evolutionarily divergent of the four families (Fig. 1d). These observations indicate that the evolutionary speed of each gene family was different, but their divergence patterns were similar.

Phylogenetic reconstruction using the neighbor-joining (NJ) method showed that the members of MAGO protein family from animal, algal and angiosperm lineages were clustered into three respective groups along the phylogenetic tree (Fig. 1a). In the angiosperm lineage, the MAGO members of monocots and dicots were divided into two subgroups. PFMAGO1 and PFMAGO2 were clustered into one group with homologous members from solanaceous species, while PFMAGO1 was relatively close to solanaceous MAGO proteins (Fig. 1a). The MAGO members from the two species of algae were clustered and located at the base of the angiosperm-lineage, but with longer branch lengths (Fig. 1a). Similar topological structures were found in each phylogeny of Y14, eIF4AIII and BTZ protein families (Fig. 1b–d), indicating that they might have undergone similar evolutionary histories.

### The expression of EJC core genes in *Physalis*

**Fig. 2:**
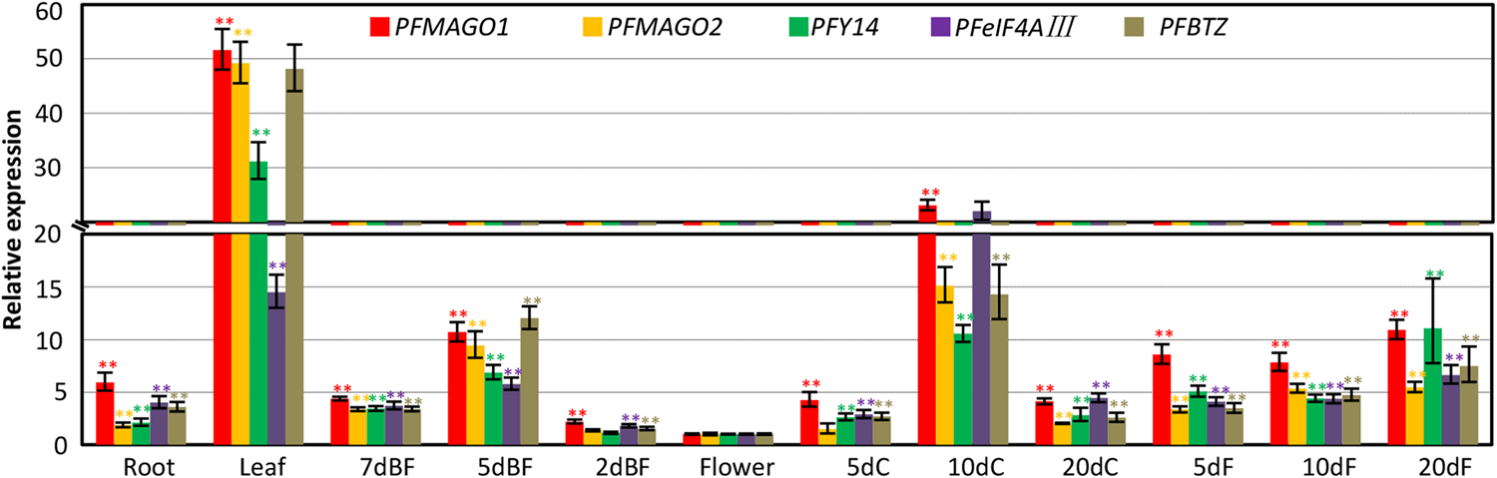
Expression patterns of EJC core genes in *P. floridana*. The total RNAs from roots, leaves, flower buds (7dBF, 5dBF, and 2dBF abbreviated for the flower buds of 7-, 5-, and 2-day before flowering), blooming flowers, fruiting calyx (5dC, 10dC and 20dC is short of the calyx of 5-, 10- and 20-day after fertilization) and developing berries (5dF, 10dF and 20dF represent fruits of 5-, 10- and 20-day after fertilization) were subjected to qRT-PCR. *PFACTIN* was used as an internal control. Experiments were performed using three independent biological samples. Mean and standard deviation are presented. The significance of gene expression differences in the detected organs relative to flowers was evaluated by a two-tailed *t*-test. Double asterisks in the same color for the same gene indicates significance at *P* < 0.01

To obtain functional clues, gene expressions of *Physalis* EJC core genes were investigated. Semi-quantitative RT-PCR was first performed with total RNA from roots, leaves, floral buds, flowers, fruiting calyx, and berries of *P. floridana*. The results indicated that five genes (*PFMAGO1, PFMAGO2, PFY14, PFeIF4AIII* and *PFBTZ*) were all transcribed, but at different levels, in the organs investigated (Supplementary Fig. S7). To quantify gene expression levels in each organ, qRT-PCR assays were performed. Gene expression data showed that each of the five genes had variable expression levels in different organs, and all five genes were also divergent within the same organs. Nonetheless, these genes shared an overall similar expression pattern (Fig. 2). The lowest expression of all genes was found in mature flowers. The expression of all genes was significantly higher in other detected organs and, in particular, they were highly expressed in leaves and 10 d old fruiting calyces (Fig. 2). Moreover, the expression of each gene was elevated in developing berries (Fig. 2). The extensive expression in all organs and the coincident expression profiles of these genes hinted that the EJC core component may cooperatively play multiple roles in growth, development and reproduction in *P. floridana*.

### Subcellular localization of *Physalis* EJC core proteins

**Fig. 3:**
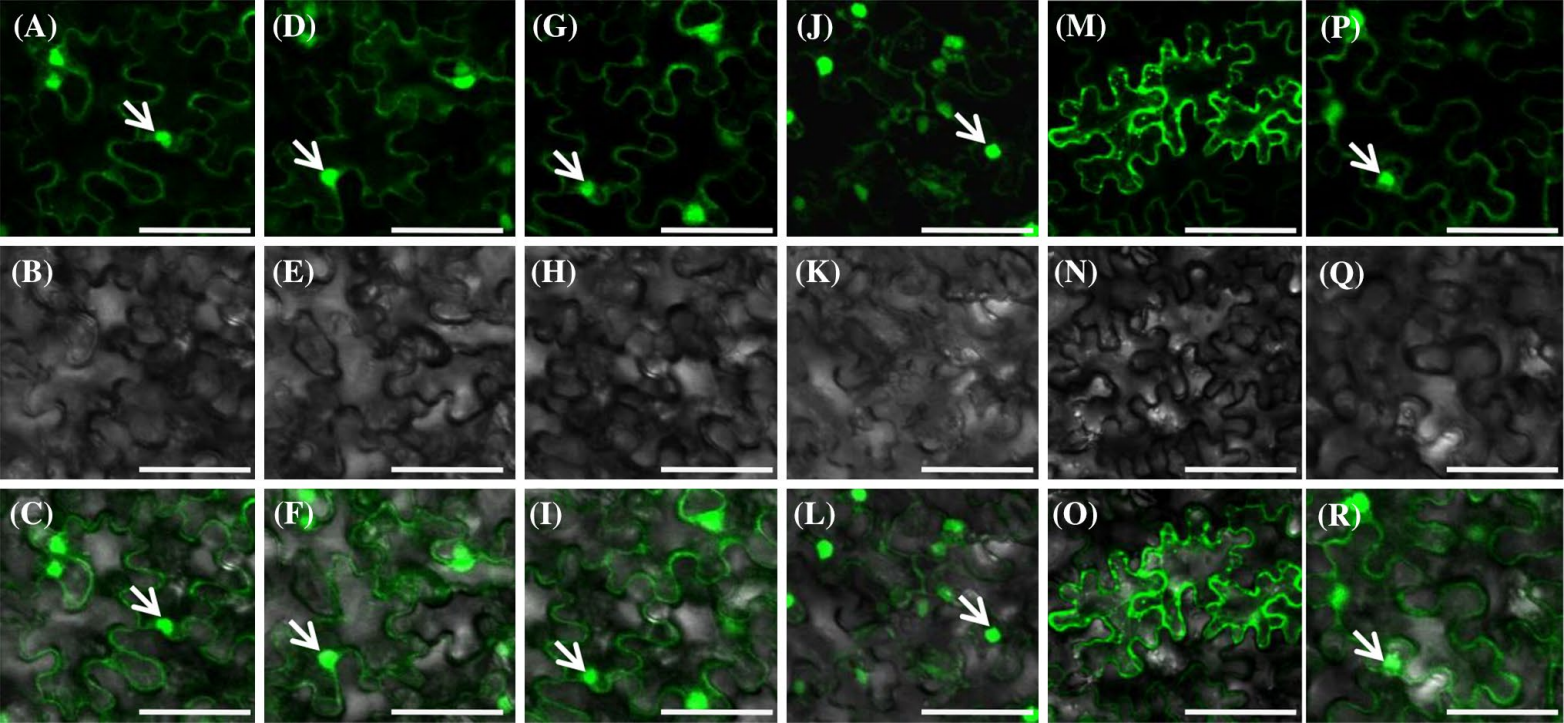
Subcellular localization of *Physalis* EJC core proteins in plant cells. **a**–**c** PFMAGO1-GFP. **d**–**f** PFMAGO2-GFP. **g**–**i** PFY14-GFP. **j**–**l** PFeIF4AIII-GFP. **m**–**o** PFBTZ-GFP. **p**–**r** GFP protein as the control. Bars, 50 µm. The first row shows signals in fluorescence fields; the second row shows signals in bright fields; the third row shows merged signals of fluorescence and bright fields. The arrow indicates the nucleus

The EJC core proteins in animals are nucleocytoplasmic shuttling proteins (Le Hir et al. 2016). To study subcellular localization patterns of *Physalis* EJC core components, the ORF of each gene was fused in frame with GFP and was transiently expressed in tobacco leaf cells. The distribution of the GFP expression signal of the resultant construct in leaf cells indicated the subcellular localization of the EJC proteins. Using this technique, we found that PFMAGO1 (Fig. 3a–c), PFMAGO2 (Fig. 3d–f), PFY14 (Fig. 3g–i), and PFeIF4AIII (Fig. 3j–l) shared a similar distribution in that they might be localized both in the nucleus and cytoplasm. However, PFBTZ showed a distinct localization pattern. It was exclusively localized in cytoplasm, particularly in some small bodies (Fig. 3m–o). The GFP protein, as a control, was localized both in the nucleus and the cytoplasm (Fig. 3p–r). These results indicate that the *Physalis* EJC core may function in the nucleus and cytoplasm or have nucleocytoplasmic shuttling potential.

### PPIs among *Physalis* EJC core components

Animal EJC core components form a tetramer to perform biological functions (Le Hir et al. 2016). Therefore, we studied the PPI capability among the *Physalis* EJC core components. A yeast two-hybrid assay was initially used (see “Materials and methods” section). MPF2 interacts with PFMAGO1 and PFMAGO2 (He et al. 2007) and thus these PPIs were included as the positive controls. An empty prey or a bait vector was included as a negative control. No interaction was observed between the *Physalis* proteins with the empty vector controls since there was no cell growth and no β-galactosidase activity (blue coloration). However, several PPIs among the *Physalis* EJC proteins were detected based on cell growth and appearance of the blue coloration (Fig. S8). Consistent with previous work (He et al. 2007), MPF2 formed homodimers and it also interacted with PFMAGO1 and PFMAGO2 (Fig. S8). However, MPF2 did not interact with the other *Physalis* EJC cores. Among the EJC, the PFY14 interacted with PFMAGO1 or PFMAGO2 protein (Fig. S8). PFBTZ might form homodimers since the yeast cells could grow normally, albeit with extremely low β-galactosidase activity. PFBTZ heterodimerized with PFMAGO1 or PFMAGO2 when PFBTZ acted as a prey protein. Interestingly, PFeIF4AIII did not interact with any of these EJC core components in yeast (Fig. S8).

**Fig. 4:**
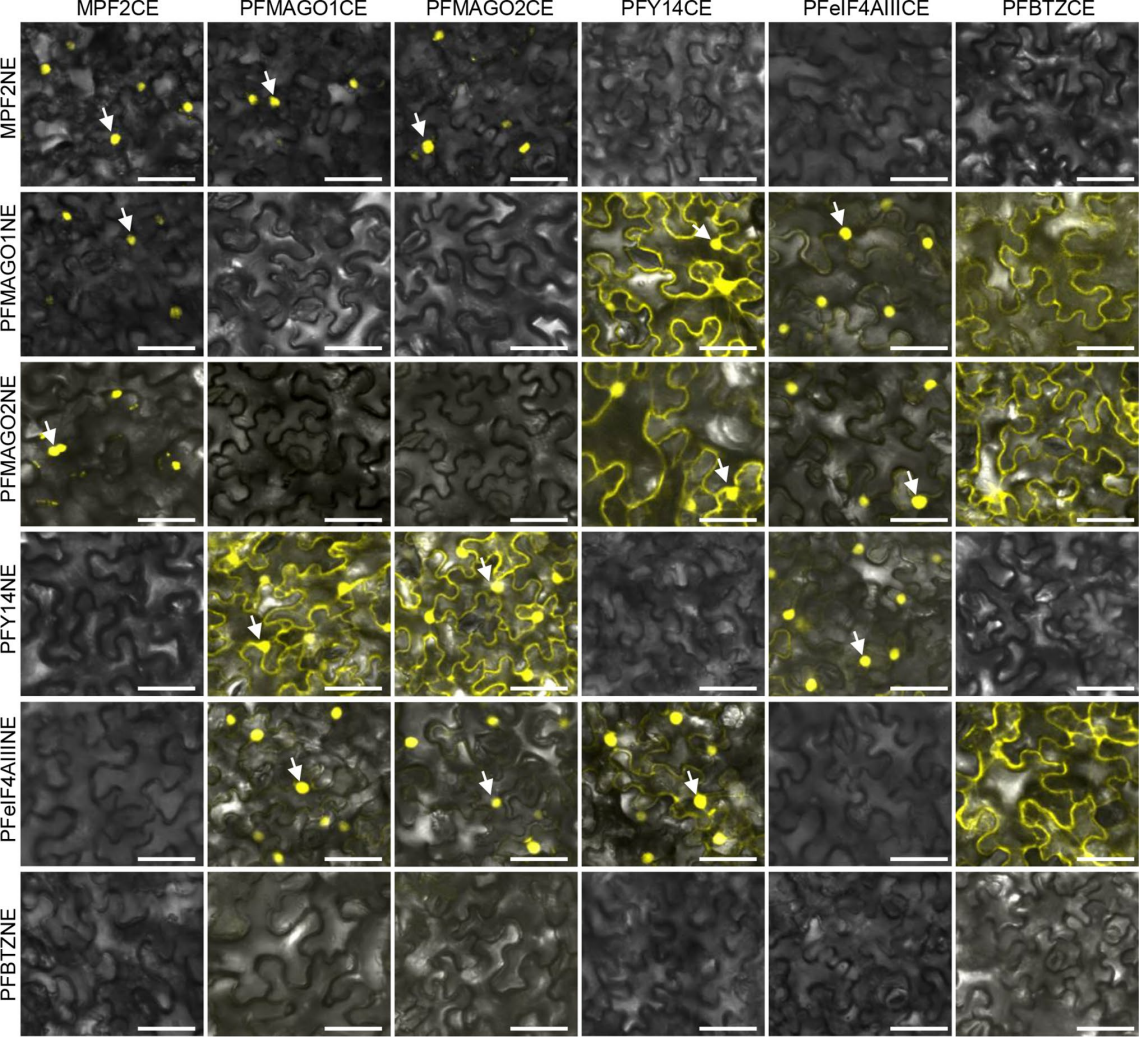
PPIs of *Physalis* EJC core in plant cells. Tobacco epidermal cells were co-transformed with the constructs of both YFPn (vertical panels) and YFPc (horizontal panels) fusion proteins. The arrow indicates the nuclei. Bars, 50 µm

The PPIs among *Physalis* EJC core proteins were also investigated in plant cells by bimolecular fluorescence complementation (BiFC) analyses. The ORF of each EJC core protein was cloned into a *pSPYNE-35S* or *pSPYCE-35S* vector that coded half of the yellow fluorescence protein (YFP) to generate EJC-YFPn or EJC-YFPc fusion proteins, respectively. The detection of the YFP signal in plant cells that were co-transformed with any combination of YFPn- and YFPc-derived fusion constructs indicated that the two proteins could interact. Otherwise, no PPI was detected. In BiFC assays, the positive control, MPF2 not only formed homodimers but also interacted with each of the MAGO (PFMAGO1 and PFMAGO2) proteins, and the YFP signals clearly originated in the nucleus (Fig. 4). Moreover, MPF2 did not interact with other EJC cores in plant cells (Fig. 4). Among the EJC core components, PFMAGO1 or PFMAGO2 interacted with PFY14 both in the nucleus and cytoplasm to form heterodimers. As such, the detected PPIs and non-PPIs patterns in plant cells (Fig. 4) were similar to the Y2H assay (Supplementary Fig. S8). However, some differences were apparent. In plant cells, the PFeIF4AIII could interact with the other three EJC core components (PFMAGO1, PFMAGO2 and PFY14) to form different heterodimers, and strong YFP signals in the nucleus were observed (Fig. 4). In plant cells PFBTZ was unable to form the homodimer but additional heterodimerization with PFeIF4AIII in one orientation was detected and all the detected PPI (YFP) signals associated with PFBTZ were largely from the cytoplasm, i.e., when heterodimerizing with PFMAGO1, PFMAGO2, and PFeIF4AIII (Fig. 4).

### Developmental roles of EJC core components in *Physalis*

To study developmental roles of *PFMAGO1, PFMAGO2, PFY14, PFeIF4AIII* and *PFBTZ* in *P. floridana*, we used the virus-induced gene silencing (VIGS) approach. A gene-specific coding fragment was used as the trigger sequence to create VIGS constructs (*PFMAGO1*-*TRV2, PFY14-TRV2, PFeIF4AIII-TRV2*, and *PFBTZ-TRV2*) (Supplementary Fig. S1). One hundred and twenty *P. floridana* seedlings (30 days old) were infected for each gene. A total of 30 seedlings were simultaneously infected using an empty *TRV2* construct as the negative control (NC) and 30 seedlings were untreated as wild type (WT) controls (Supplementary Fig. S9a). It was difficult to make a gene-specific silencing construct due to high sequence identity (87.6%) of *PFMAGO1* and *PFMAGO2*. Thus, the two genes were aimed to be simultaneously downregulated using part of *PFMAGO1* as the trigger sequence, which shared 89.0% sequence identity with the corresponding section of *PFMGO2* (Supplementary Fig. S1). The derivative VIGS plants were named *PFMAGO*-VIGS. The VIGS products of other genes were respectively named as *PFY14-, PFeIF4AIII*- and *PFBTZ*-VIGS plants. The expression of each EJC gene in *Physalis* leaves was detected in VIGS plants using qRT-PCR analyses at 14 days after infection (14DAI) and revealed 112–115 true downregulated VIGS plants for each gene (Supplementary Fig. S9a). These VIGS plants grew as normal as the WT and NC plants before the 14DAI stage (Supplementary Fig. S9b). However, more than half of *PFY14*- and *PFMAGO*-VIGS plants, respectively, began to display abnormal growth at the 21DAI and 28DAI stages, while the *PFeIF4AIII*- and *PFBTZ*-VIGS plants were apparently normal and similar to the WT and NC plants at these stages (Supplementary Fig. S9b). Further analyses of the results are described, below, per gene.

### PFMAGO genes have roles in both vegetative and reproductive growth

**Fig. 5:**
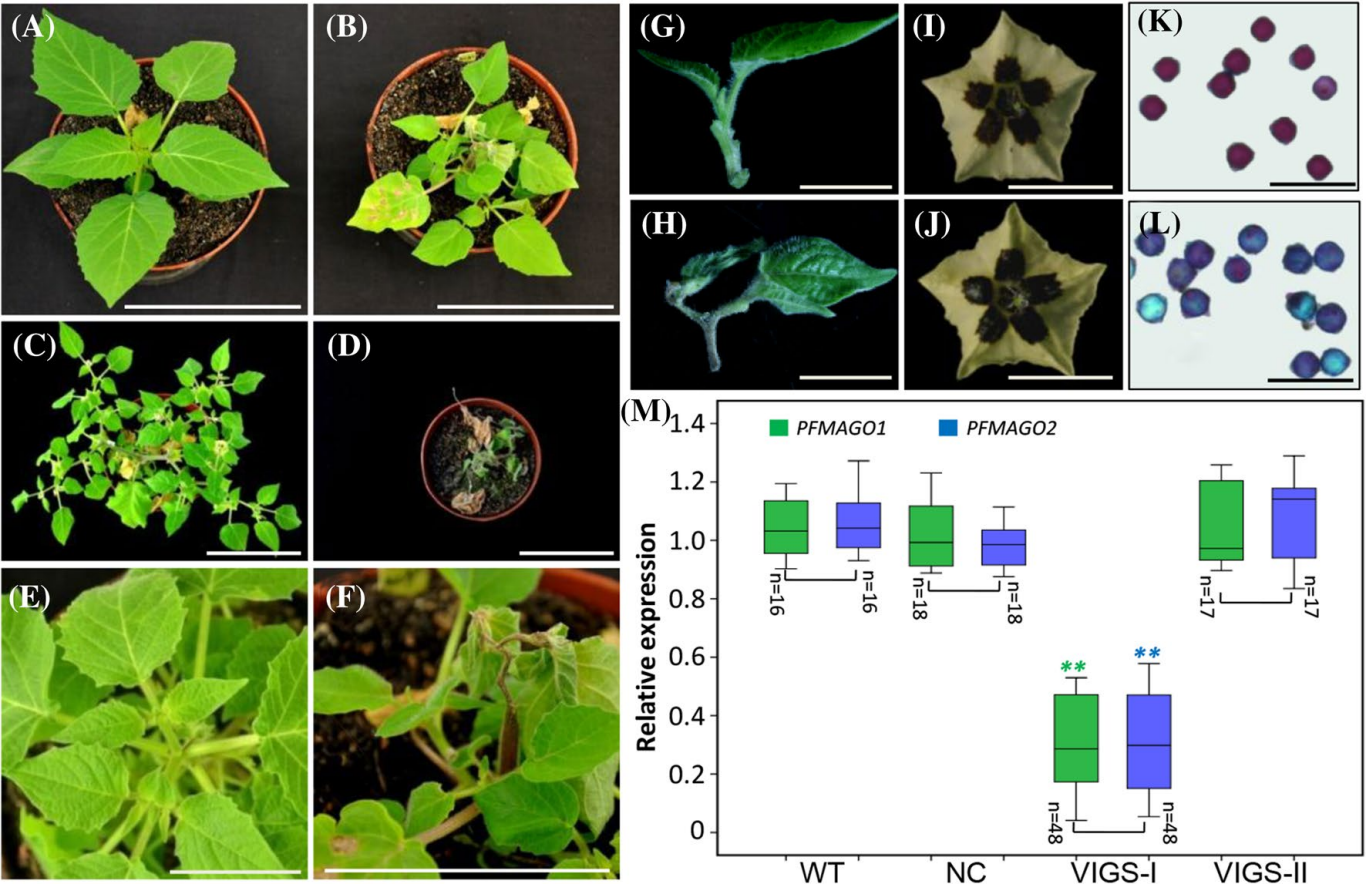
Phenotypic analysis of *PFMAGO*-VIGS in *P. floridana*. **a, b** NC and *PFMAGO*-VIGS plants in 28DAI stage. **c, d** NC and *PFMAGO*-VIGS plants in the fruiting stage. **e, f** Phenotype of shoot apex growth at 28DAI stage in NC and *PFMAGO*-VIGS plants. **g, h** Shoot apex of NC and *PFMAGO*-VIGS plants. **i, j** NC and *PFMAGO*-VIGS flowers. **k, l** Pollen viability in NC and *PFMAGO*-VIGS flowers, which was evaluated by I_2_–KI staining. **m** The expression of both *PFMAGO1* and *PFMAGO2*. Total RNAs from different flowers and number (n) as indicated were subjected to qRT-PCR. *WT* wild type, *NC* negative control, VIGS-I, *PFMAGO*-VIGS flowers showing pollen abortion; VIGS-II, *PFMAGO*-VIGS flowers showing WT-like pollen development. Bars, 10 cm in **a**–**h**, 1 cm in **i** and **j**, and 100 µm in **k, l**. Significance relative to NC was evaluated by a two-tailed student’s *t* test, and double asterisks indicates significance at *P* < 0.01

*PFMAGO*-VIGS plants grew normally before 21DAI (Supplementary Fig. S9b) then leaves at the top of the main stem became warped and drooped compared to NC and WT (Fig. 5a, b). Three months later, NC and WT plants had luxuriant growth with many flowers and fruits (Fig. 5c), but about 75% of the *PFMAGO*-VIGS plants had withered and died after flowering (Fig. 5d). Shoot apex exsiccation and leaf withering might have been the main causes of plant death (Fig. 5e–h). Only 25 out of 115 (21.7%) VIGS plants survived (Supplementary Fig. S9a). These surviving plants attained the reproductive stage and developed a few flowers. Compared to WT and NC, flower morphology in *PFMAGO*-VIGS plants did not appear to be changed (Fig. 5i, j). However, only rarely did flowers produce fruits and the fruit setting rate was 35.3% (Supplementary Fig. S9a). If cross-pollinated with WT pollen, the fruit setting reached 90% (Supplementary Fig. S9a), indicating that the female organ was functional. However, I_2_–KI staining assays revealed that pollen viability or maturation of most *PFMAGO*-VIGS flowers was greatly reduced compared to NC (Fig. 5k, l and Supplementary Fig. S9a). qRT-PCR analyses showed that the expression of both *PFMAGO* genes was significantly knocked down in these *PFMAGO*-VIGS flowers (*P* < 0.01, defined as VIGS-I). In the *PFMAGO*-VIGS flowers that showed WT-like pollen development (defined as VIGS-II), the *PFMAGO* expression was not altered compared to the WT and NC plants (Fig. 5m). These results indicated that *PFMAGO* genes in *P. floridana* primarily affect shoot apex development and male fertility.

### PFY14 is involved in shoot apex development and fertility processes

*PFY14*-VIGS plants grew normally before the 14DAI stage but they began to differ from WT and NC plants at the 21DAI stage (Supplementary Fig. S9b and Supplementary Fig. S10a, b). Some lines of *PFY14*-VIGS plants displayed early blossoming and displayed a severe leaf withered phenotype (Supplementary Fig. S10b). Three months later, all *PFY14* effectively downregulated VIGS plants were dead (Supplementary Fig. S9a). During the vegetative growth stage, the shoot apex of WT and NC plants was deep green and viable (Supplementary Fig. S10c). In *PFY14*-VIGS plants the shoot apex was distorted and showed a leaf-blight-like phenotype (Supplementary Fig. S10d) with inhibition of new leaf or flower primordia morphogenesis. Fewer and smaller flowers relative to WT and NC were observed in some *PFY14*-VIGS plants (Supplementary Fig. S10b, e, f). Unfortunately, these flowers quickly withered and dropped after bloom. Pollen viability was investigated, compared to NC. Less than 36% of the pollen grains from the *PFY14*-VIGS flowers stained blue (Supplementary Figs. S9a, S10g, h), suggesting inhibition of pollen maturation. At the end of the experiment, no fruit was obtained from the *PFY14*-VIGS plants either naturally or by artificial pollination with WT pollen (Supplementary Fig. S9a). This indicated that the female function might also have been damaged. The *PFY14* expression in all observed *PFY14*-VIGS flowers was seriously down-regulated compared to WT and NC plants (*P* < 0.01, Supplementary Fig. S10i). Therefore, *PFY14* plays significant roles in shoot apex development, carpel functionality and male fertility processes in *P. floridana*.

### PFeIF4AIII roles in Chinese lantern development and root collar growth

**Fig. 6:**
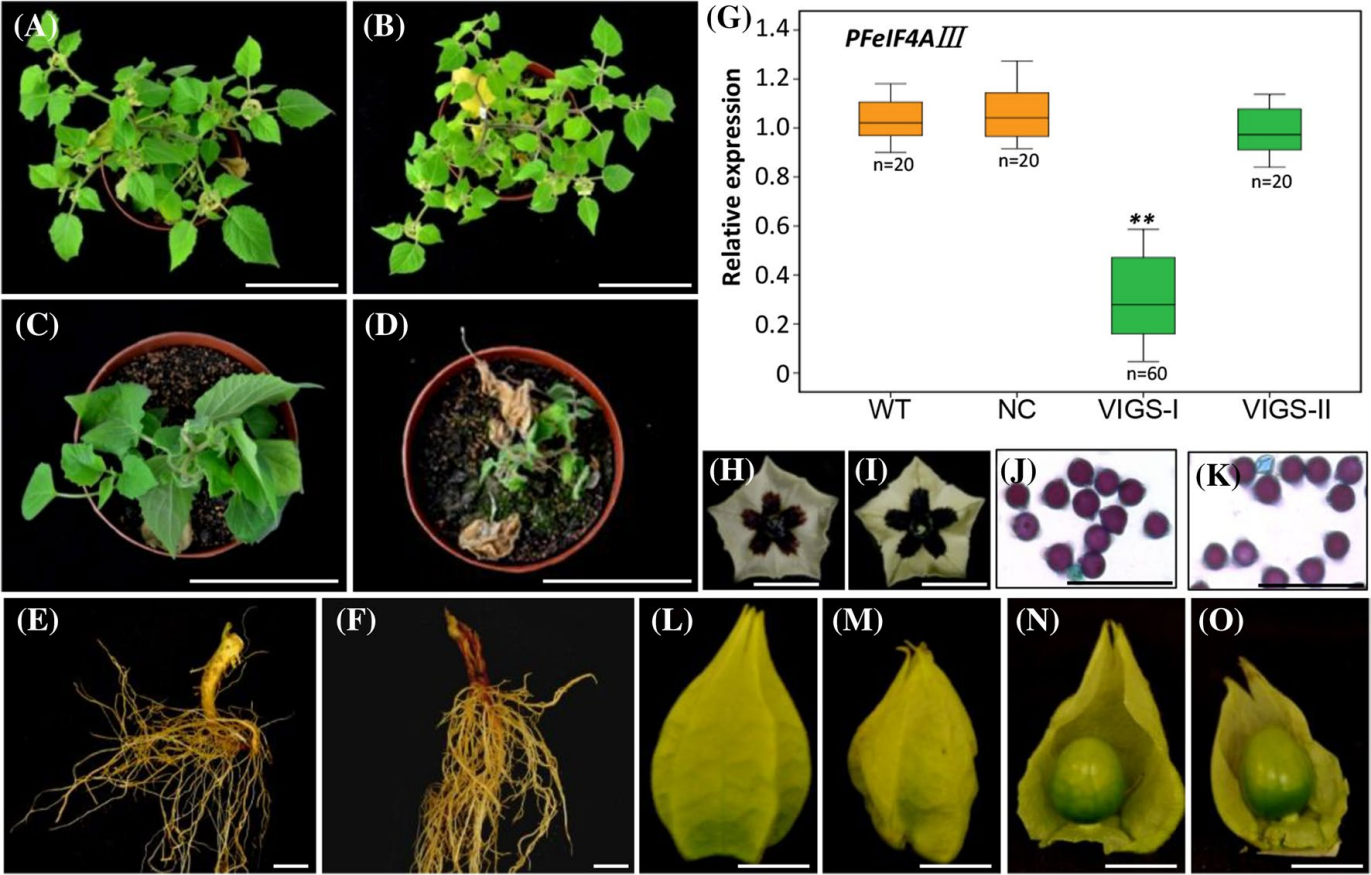
*PFeIF4AIII*-VIGS analysis in *P. floridana*. **a, b** Growth of NC and *PFeIF4AIII*-VIGS plants at the fruiting stage. **c, d** *PFeIF4AIII*-VIGS plants displayed wilting and died after flowering compared to NC plants. **e, f** Root growth of NC and *PFeIF4AIII*-VIGS plant. **g** The *PFeIF4AIII* expression in root collar area of plants as indicated. WT, wild type; NC, negative control; VIGS-I, *PFeIF4AIII*-VIGS plants showing root collar decay; VIGS-II, *PFeIF4AIII*-VIGS plants having WT-like root collar. The investigated sample number (n) in each case was indicated. **h, i** Flower morphology in NC and *PFeIF4AIII*-VIGS plants. **j, k** Pollen viability in NC and *PFeIF4AIII*-VIGS flowers, which was evaluated by I_2_–KI staining. **l, m** ICS morphology in NC and *PFeIF4AIII*-VIGS plants. **n, o** Fruit morphology in NC and *PFeIF4AIII*-VIGS plants. Bars, 10 cm in **a-d**, 2 cm in **e, f**, 1 cm in **h, i** and **l**–**o**, and 100 µm in **j, k**. Significance relative to NC was evaluated by a two-tailed student’s *t* test, and double asterisks indicates significance at *P* < 0.01

Nearly 40% (44 out of 113) *PFeIF4AIII*-VIGS grew as strongly as NC plants at the flowering stage, and these plants blossomed and bore fruit normally (Fig. 6a, b and Supplementary Fig. S9a). However, the other 69 *PFeIF4AIII*-VIGS plants died after flowering (Fig. 6c, d). We found that the root collar area was rotten in these dead *PFeIF4AIII*-VIGS plants compared to the NC plants (Fig. 6e, f). qRT-PCR results indicated that *PFeIF4AIII* expression in the root collar of these *PFeIF4AIII*-VIGS plants was severely downregulated (*P* < 0.01, defined as VIGS-I), while, in the *PFeIF4AIII*-VIGS plants that showed normal phenotypes (defined as VIGS-II), the gene expression was not altered compared to the WT and NC plants (Fig. 6g). Flower morphologies and pollen grain activities of the surviving *PFeIF4AIII*-VIGS plants showing severe *PFeIF4AIII* downregulation was similar to NC plants (Fig. 6h–k), and these plants could set fruit naturally or via artificial pollination with WT pollen (Supplementary Fig. S9a). The Chinese lantern shape of *PFeIF4AIII*-VIGS plants was irregular compared to NC (Fig. 6l, m) but the berries were normal (Fig. 6n–o). Therefore, *PFeIF4AIII* may be involved in Chinese lantern development and root collar growth in *P. floridana*.

### PFBTZ is mainly involved in male fertility

*PFBTZ*-VIGS plants differed from *PFMAGO*-, *PFY14*-, and *PFeIF4AIII*-VIGS plants and they appeared as normal as WT or NC plants during the entire developmental process (Supplementary Fig. S9 and Supplementary Fig. S11a, b). All of the *PFBTZ*-VIGS plants survived and set fruits (Supplementary Fig. S9a). The morphology of the flowers and fruits of the *PFBTZ*-VIGS plants was similar to NC plants (Supplementary Fig. S11c–f). The fruit setting rate of the *PFBTZ*-VIGS plants under natural conditions was only 68% and this was significantly less than NC plants (Supplementary Fig. S9a). However, artificial pollination with WT pollen significantly increased the fruit setting rate to 92% (Supplementary Fig. S9a), suggesting that the female organ was not affected. I_2_–KI assays showed that pollen grain viability in *PFBTZ*-VIGS flowers was poor (~ 50%) compared to WT and NC flowers (Supplementary Figs. S9a, S11g, h). The *PFBTZ* expression in these mutated flowers was significantly downregulated (VIGS-I) compared to WT and NC (*P* < 0.01), while pollen viability of the *PFBTZ*-VIGS plants that showed the *PFBTZ* expression of VIGS-II grade was normal (Supplementary Fig. S11i). Thus, *PFBTZ* mainly participates in the functional determination of male fertility in *P. floridana*.

### Gene expression variation in knockdowns of *P. floridana* EJC core genes

#### EJC controls the splicing of *P. floridana* dysfunctional tapetum1 (PFDYT1)

**Fig. 7:**
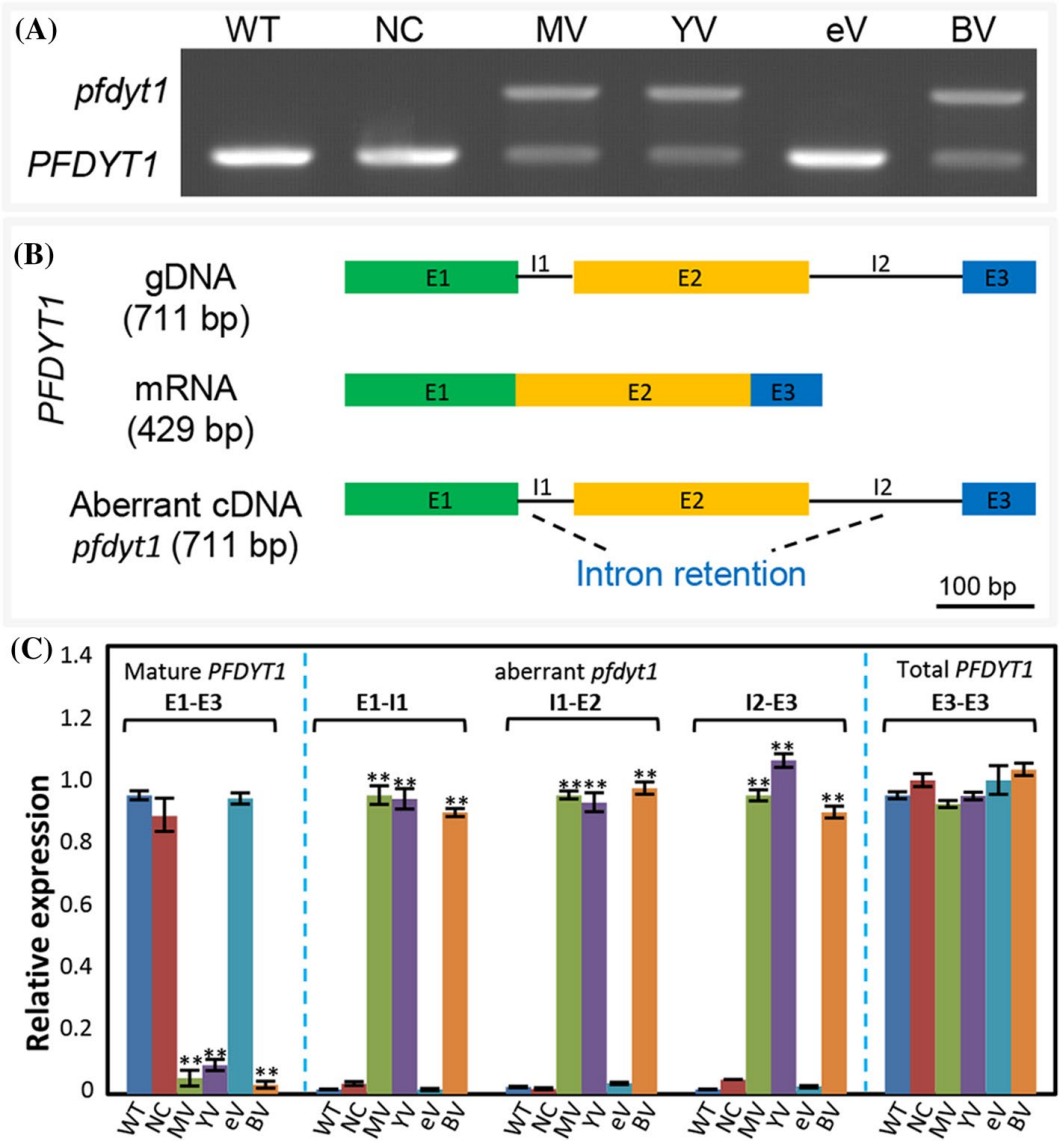
Accumulation of aberrant *pfdyt1* transcripts in EJC core knockdowns. **a** Full-length transcripts of *PFDYT1* in floral buds revealed by RT-PCR. WT, NC, MV, YV, eV and BV respectively represent wild type, negative control (*TRV2*), *PFMAGO*-, *PFY14*-, *PFeIF4AIII*- and *PFBTZ*-VIGS plants. The extension time was 30 s. A larger transcript designated *pfdyt1* occurred in the indicated flowers. **b** The *PFDYT1* splicing is altered in the knockdowns of the EJC core genes. The structure of gDNA, cDNA in WT plants, and aberrant transcript in the VIGS plants was demonstrated. Color rectangles, exons (E1–E3); black lines, introns (I1, I2). **c** Relative expression levels of mature *PFDYT1*, abnormal *pfdyt1* (E1–I1, I1–E2, I2–E3) and total (E3–E3) mRNA as indicated. Total RNAs from floral buds (7DBF) of WT, NC and *PFMAGO*-, *PFY14*-, *PfeIF4AIII*- and *PFBTZ*-VIGS plants were subjected to qRT-PCR. *PFACTIN* mRNAs were used as internal control. The extension time was 10 s. In detection of mature *PFDYT1* RNA (left), the expression in WT was set as 1, while in detection of aberrant *pfdyt1* transcripts (right), the expression in floral buds of each MV was set as 1. Three independent biological samples were used, and error bars represent SD. Significance relative to WT was evaluated by a two-tailed student’s *t* test, and double asterisks indicates significance at *P* < 0.01

A common abnormality in *PFMAGO*-, *PFY14*-, and *PFBTZ*-VIGS plants was to produce premature pollen. We therefore detected the expression variation of the genes involved. The splicing of the *OsUDT1* gene that is essential to pollen maturation in rice is regulated by the EJC core (Gong and He 2014; Huang et al. 2016). Two orthologs of the *OsUDT1* gene, tomato *male sterile 10*^*35*^ (*Ms10*^*35*^) and *Arabidopsis AtDYT1*, also play essential roles in pollen development and meiosis in anthers (Jeong et al. 2014). Using *Ms10*^*35*^ (XM_004233756) as the template to screen the *P. floridana* transcriptome, only one unigene was found with high sequence identity (91.6%), and this was named *P. floridana dysfunctional tapetum 1* (*PFDYT1*). The ORF of *PFDYT1* was 429 base pairs. To investigate expression variation of the *PFDYT1* gene, total RNAs of floral organs were extracted from the WT, NC, *PFMAGO*-, *PFY14*-, *PFeIF4AIII*- and *PFBTZ*-VIGS plants and subjected to conventional RT-PCR assays (Fig. 7a). RT-PCR, using gene-specific primers that flanked the full coding region, showed that the mature *PFDYT1* transcripts were severely downregulated in *PFMAGO*-, *PFY14*- and *PFBTZ*-VIGS flowers, but a large transcript of about 700 bp was also detected compared to WT, NC and *PFeIF4AIII*-VIGS plants (Fig. 7a). Cloning and sequencing showed that the large *PFDYT1* transcript was 711 bp and was identical with its genomic sequence including three exons and two introns (Fig. 7b). This suggested that intron retention happened during *PFDYT1* transcription once *PFMAGO, PFY14* or *PFBTZ* was downregulated. The aberrant transcripts (*pfdyt1*) due to intron retention failed to encode full-length PFDYT1 proteins (Supplementary Fig. S12). To rigorously confirm the expression variation, qRT-PCR was conducted. The variation of *PFDYT1* mature mRNA level, which significantly decreased (*P* < 0.01, Fig. 7c), was identical to the observation from routine RT-PCR (Fig. 7a). However, when a primer flanking occurred on the intron (i.e., E1–I1, I1–E2, or I2–E3), a contrasting variation was observed, and strong expression signal of the *PFDYT1* transcripts occurred in *PFMAGO*-, *PFY14-*, and *PFBTZ*-VIGS floral organs instead of WT, NC and *PFeIF4AIII*-VIGS flowers (Fig. 7c), indicating an increase of aberrant *pfdyt1* transcripts in *PFMAGO*-, *PFY14-, PFBTZ*-VIGS flowers, while the total mRNA level seemed not to be affected. We further investigated this using primers flanking exon3 (E3), which were presumed to amplify the total *PFDYT1* mRNA level. It turned out that the total mRNA level of *PFDYT1* gene was indeed comparable between the controls and the VIGS plants (Fig. 7c), suggesting that the unspliced forms of the *PFDYT1* mRNA in these VIGS plants were stably accumulated. Therefore, *PFMAGO, PFY14* and *PFBTZ* are involved in the control of the splicing of the *PFDYT1* transcripts.

#### EJC alters gene expression levels of tapetal programmed cell death pathway

A well-studied pathway is the function of male fertility associated with the tapetal programmed cell death (PCD) pathway, forming *DYT1*-*TDF1*-*AMS*-*bHLH91* transcriptional cascade in rice, *Arabidopsis* and tomato (Jeong et al. 2014). We investigated the expression of the downstream genes of *PFDYT1* in *P. floridana*. Tomato (*Solanum lycopersicon*) tapetal PCD genes *SlTDF1* (*Soly03g113530*), *SlAMS* (*Soly08g062780*), *SlbHLH91* (*Soly01g081100*), *SlCysteine protease* (*SlCysP, Soly07g053460*) and *SlAspartic protease* (*SlAspP, Soly06g069220*) were used to blast the *P. floridana* transcriptome, revealing their putative orthologs named *PFTDF1* (MH319843), *PFAMS* (MH319844), *PFbHLH91* (MH319845), *PFCysP* (MH319846), and *PFAspP* (MH319847). Their mature transcripts were amplified using RT-PCR, and transcript form was not altered compared to WT and NC (Supplementary Fig. S13a). However, the expression of *PFTDF1, PFAMS, PFbHLH91*, and *PFCysP* was downregulated in *PFMAGO*-, *PFY14* and *PFBTZ*-VIGS flowers (*P* < 0.01, Supplementary Fig. S13a, b). Meanwhile, the expression of *PFAspP* in *PFMAGO*- and *PFY14*-VIGS flowers was downregulated but not affected in the *PFBTZ*-VIGS flowers (*P* < 0.01, Supplementary Fig. S13a, b). Nevertheless, the expression of all investigated genes was not altered in *PFeIF4AIII*-VIGS flowers (Supplementary Fig. S13a, b). Thus, *PFMAGO, PFY14* and *PFBTZ* may affect gene expression of microspore development pathways in *P. floridana*.

#### EJC affects expression level of WRKY genes associated with defense-related pathways

Plant death was observed in the knockdowns of some *Physalis* EJC core genes, and either withered leaves or rotten root collar phenotypes were seen in these plants. These responses might be an alteration in the resistance or defense to certain biotic stresses. To study this, we also detected the expression of relevant genes. The *WRKY* genes *StWRKY8* and *StWRKY1* in potato, *PtWRKY70* in *Populus*, and their tomato orthologs *SlWRKY31, SlWRKY75* and *SlWRKY81* are involved in resistance to the plant leaf blight phenotype (Mandal et al. 2015; Yogendra et al. 2015, 2017). We therefore isolated the three *Physalis WRKY* genes named *PFWRKY31* (MH319849), *PFWRKY75* (MH319850), and *PFWRKY81* (MH319851), and the expression of these *WRKY* genes in the VIGS tissues was investigated. No splicing alteration was observed but a decrease of the *WRKY* genes was detected (Supplementary Fig. S13c). Further qRT-PCR showed that *PFWRKY75* and *PFWRKY81* were severely downregulated in the shoot apex of *PFMAGO*- and *PFY14*-VIGS plants, and *PFWRKY31* and *PFWRKY81* were downregulated in the root collar of the *PFeIF4AIII*-VIGS (*P* < 0.01, Supplementary Fig. S13d), where significant plant death was observed. However, significant downregulation of only one gene *PFWRKY31* and unaltered expression of all three *WRKY* genes were respectively observed in the shoot apex of *PFBTZ-* and *PFeIF4AIII*-VIGS plants (Supplementary Fig. S13d) where no withered leaves were observed. Therefore, EJC core genes may be involved in defense-related processes associated with *WRKY* genes.

## Discussion

**Fig. 8:**
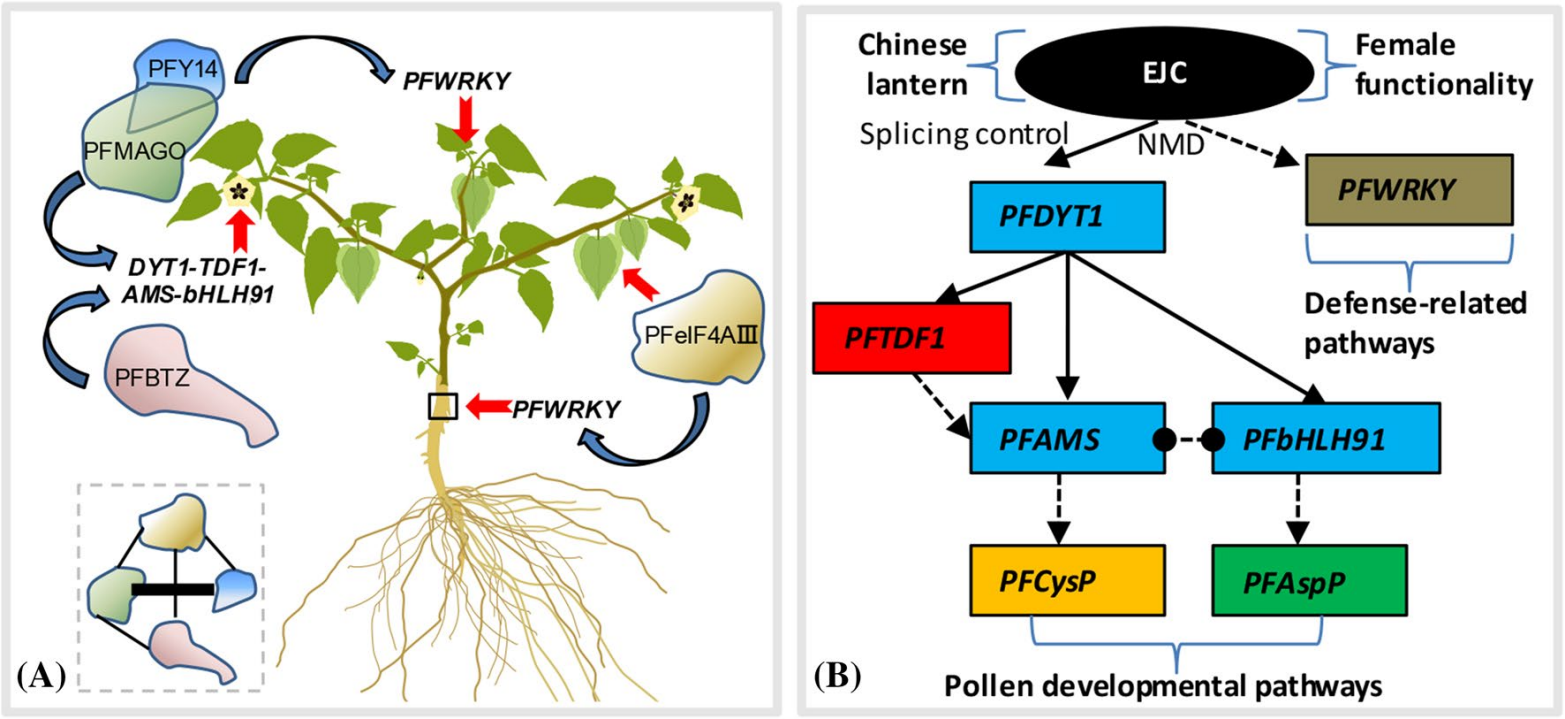
Multiple roles of EJC core homologs in *Physalis*. **a** Developmental roles of EJC core components in *Physalis*. Heterodimerization of EJC core proteins and subsequently recruiting peripheral proteins to form a functional higher order complex is essential for EJC core functions (Le Hir et al. 2016). Moreover, the MGAO-Y14 heterodimerization is obligate and functions as a functional unit (Gong et al. 2014a, b). As demonstrated in the dashed box, heterodimerization was usually formed among *Physalis* EJC core proteins (indicated by the line), but heterodimerization of PFY14 and PFBTZ was not observed. The obligate PFMGAO-PFY14 heterodimerization is highlighted using the thick line. Single *Physalis* EJC core protein or heterodimer PFMGAO-PFY14 is presented to stand for each-containing functional complex instead of itself. Blue arrows indicate activation of the downstream genes, and red arrows point to the organs or tissues where the EJC genes exert their developmental affects. Black box defines the root collar area. For the details, see text. **b** The proposed working model for EJC genes in *Physalis*. The putative tapetal PCD genes in *Physalis* were constructed according to Jeong et al. (2014). Blue boxes, basic helix-loop-helix (bHLH) transcription factor; red box, MYB transcription factor; yellow box, cysteine (Cys) protease gene; green box, aspartic (Asp) protease gene; gray box, WRKY transcription factor. The dot indicates PPI. Arrows represent positive regulation. The dashed lines represent predicted regulation or interaction

Molecular evolution of EJC core genes is conserved, but their developmental roles seem to be diverse. The EJC core genes have been well studied in animals. However, studies of the protein core complex in plants have been less thorough. In this study, we compared the evolutionary conserved characteristics of *P. floridana* EJC core genes to other orthologs and found that they are involved in multiple developmental processes. Reduced fruit setting after downregulating EJC core genes appeared to be a consequence of poor fertility, particularly male fertility, since artificial fertilization with WT pollen largely rescued fruit setting. Therefore, EJC core genes are primarily involved in male fertility pathways and may participate in female functionality determination, Chinese lantern development or defense-related processes in *P. floridana* (Fig. 8a).

### Functional conservation of plant EJC genes in male fertility

*MAGO, Y14* and *eIF4AIII* genes have been studied in other plant species, and they are mainly involved in male fertility (He et al. 2007; van der Weele et al. 2007; Park et al. 2009; Boothby and Wolniak 2011; Gong and He 2014; Gong et al. 2014a; Ihsan et al. 2015; Cilano et al. 2016; Huang et al. 2016). However, no functional role for the *BTZ* gene has been found in plants. We found, using the VIGS approach, that *PFMAGO*s, *PFY14* and *PFBTZ* are all involved in the pollen maturation process. This suggests a conserved role of the EJC core in male fertility. However, the mechanisms of these genes in male fertility are unknown. Serving as the first functional clue, PFMAGO1 and PFMAGO2 were observed to interact with MADS-domain protein MPF2, which plays a role in male fertility (He and Saedler 2005; He et al. 2007), suggesting that PFMAGO is involved with male fertility control by interacting with the essential regulators of pollen development. Further support for this assumption comes from *Withania somnifera*, where the *WsMAGO2* gene is regulated through the anther-specific GAATTTGTGA motif, and the encoded protein interacts with MPF2-like proteins and affects male fertility by producing abortive pollen or seeds (Ihsan et al. 2015).

Rice studies have also provided clues to the role of EJC core genes in male fertility. RNAi of *OsMAGO1-OsMAGO2* and *OsY14a* made various splicing products of *OsUDT1* via intron retention and exon skipping in rice flowers. These resulted in degradation of the endothecium and tapetum leading to abnormal pollen grain development (Gong and He 2014). Identical alternative splicing evidence of *OsUDT1* was also found in *OsRH2* and *OsRH34* (*eIF4AIII* orthologs) double knockdown transgenic rice plants (Huang et al. 2016). *OsUDT1* is the ortholog of *DYT1* encoding a *bHLH* transcription factor (Jung et al. 2005; Jeong et al. 2014), which is the essential upstream regulator of the well-studied tapetum PCD pathway including the *DYT1 (bHLH)*-*TDF1 (MYB)*-*AMS (bHLH)*-*bHLH91* transcription cascade in rice, *Arabidopsis* and tomato (Jeong et al. 2014). *DYT1* is involved in tapetum development (Zhang et al. 2006; Zhu et al. 2015). *TDF1* plays a role in callose dissolution (Zhu et al. 2008). *AMS* (Sorensen et al. 2003) and *bHLH91* (Xu et al. 2010) participate in PCD-triggered cell death. The downstream genes of the transcriptional cascade *AspP* (Niu et al. 2013) and *CysP* (Lee et al. 2004; Li et al. 2006) are respectively involved in PCD-triggered cell death and in anther cell wall modification and degradation. To understand the role of the *Physalis* EJC genes in controlling male fertility, we investigated the gene expression in the tapetum PCD pathway. We determined putative orthologs of these genes in *P. floridana*. For example, *PFDYT1* is the ortholog of *OsUDT1* in rice and *SlDYT1* (*Ms 10*^*35*^) in tomato (Fig. 8b). We found that knockdown of *PFMAGO, PFY14* and *PFBTZ* genes led to abnormal *PFDYT1* pre-mRNA splicing accumulation via intron retention. Similar events were observed in the downregulation of rice EJC genes (*OsMAGO*-, *OsY14*-, or *eIF4AIII)*, and abnormal transcript species resulted from first intron retention, partial or whole exon lacking (Gong and He 2014; Huang et al. 2016). These indicate the conserved splicing target of plant EJC genes in male fertility is *DYT1* or *UDT1*.

The abnormal splicing of *PFDYT1* might affect the expression of downstream genes such as *PFTDF1, PFAMS* and *PFbHLH* (Fig. 8b), and we observed that these genes in this tapetum PCD pathway were extremely downregulated. This suggests that the transcription cascade regulating pollen development is largely conserved. Pollen development was not changed in *PFeIF4AIII*-VIGS flowers, and the expression of *PFDYT1* and the related downstream genes was not altered, indicating the target specificity of different EJC core genes. The aberrant transcript *pfdyt1* could not encode a normal PFDYT1 protein, and the downregulation of these downstream genes is likely due to reduction of mature *PFDYT1*. Therefore, downregulating any of the three *Physalis* EJC core genes (*PFMAGO, PFY14* and *PFBTZ*) disrupts the conserved EJC function in male fertility. This is correlated with the accumulation of abnormal pre-mRNA and expression alteration of a series of downstream genes in the tapetum PCD pathway, also implying that the NMD role might be impaired in the VIGS plants of these EJC core genes.

### EJC genes are involved in defense-related processes in *Physalis*

NMD is a eukaryotic quality-control mechanism that governs the stability of both aberrant and normal transcripts. NMD inhibition during biotic stress contributes to the development of immunity responses (Shaul 2015). There is evidence that EJC is involved in defense-related pathways of plants. In rice, both *OsMAGO2* and *OsY14b* genes show high sensitivity to a variety of abiotic stresses (Gong and He 2014). In *Hevea brasiliensis, HbMAGO* and *HbY14* genes have different expression patterns in response to ethylene and jasmonate treatments (Yang et al. 2016). *AteIF4AIII* shares functions in abiotic stress adaptation in *Arabidopsis*. The orthologs of the stress-related helicases PDH45 and MH1 in *Pisum sativum* and *Medicago sativa* (Pascuan et al. 2016), and subcellular localization of *Arabidopsis* eIF4AIII was influenced by hypoxic environments (Koroleva et al. 2009). These observations suggest that the EJC core component may be involved in plant defense-related pathways. In our study, phenotypes resembling leaf-blight or showing root collar decay were observed in *PFMAGO*-, *PFY14* and *PFeIF4AIII*-VIGS transgenic *Physalis* plants. We inferred that the regulation of defense against biotic stresses may be hindered due to knocking these EJC genes down.

The *WRKY* gene family in *Arabidopsis* is important in the defense-related pathways that guard against biotic stresses, such as bacterial and fungal pathogens (Ulker and Somssich 2004; Higashi et al. 2008; Kim et al. 2008; Mukhtar et al. 2008; Pandey et al. 2010). In *Solanum lycopersicon, SlWRKY* genes are also involved in response to multiple abiotic and biotic stresses including drought, salt stress and *Pseudomonas syringae* invasion (Huang et al. 2012). *SlDRW1* (*S. lycopersicon* defense-related *WRKY1*, also named *SlWRKY31*) is involved in *Botrytis cinerea* resistance and tolerance to oxidative stress in tomato (Liu et al. 2014), while the *StWRKY8* ortholog in *Solanum tuberosum* is involved in late blight resistance with the benzylisoquinoline alkaloid pathway (Yogendra et al. 2017). *StWRKY1*, the ortholog of *SlWRKY75*, confers late blight resistance by regulating phenylpropanoid metabolites in potato (Yogendra et al. 2015). *PtWRKY70*-RNAi in *Populus* alters salt stress and leaf blight disease responses (Zhao et al. 2017). We therefore studied the expression of three *WRKY* genes in *Physalis*, and found *PFWRKY31, PFWRKY75* and *PFWRKY81* were severely reduced in the shoot apex or root collar of *PFMAGO*-, *PFY14*- or *PFeIF4AIII*-VIGS plants. The growth of *PFBTZ*-VIGS plants appeared similar to wild plants, and the three *WRKY* expressions were not altered. Therefore, *PFMAGO*-, *PFY14*- and *PFeIF4AIII* might directly or indirectly regulate the expression of *WRKY* genes, via NMD for example, to affect plant defense pathways (Fig. 8b). The molecular details need further investigation, but this finding helps us to understand the adaptive role of EJC in plant evolution.

### Generality and specificity of EJC roles and targets

EJC core components MAGO, Y14, eIF4AIII and BTZ form a stable polymer structure in animals (Andersen et al. 2006; Bono et al. 2006; Gehring et al. 2009; Le Hir et al. 2016). In eukaryotes, the MAGO and Y14 protein families have undergone coevolution (Gong et al. 2014b). In this study, the similar topology of four gene families suggested that they might have undergone a similar evolutionary history. In addition, the simulated tetramer structures of the *Physalis* EJC core are conserved compared to those of animals. Therefore, EJC probably play fundamental roles in all of the eukaryotes. However, they play diverse roles in cell division, as well as physiological, developmental and adaptive roles, but were recruited in a species- or gene-specific manner (see “Introduction” section).

One possibility for the specificity in functions might involve the selective targets at transcription and different posttranscriptional levels such as localization, splicing and translation. In *Drosophila, oskar* mRNA proper localization is a primary target of EJC (Boswell et al. 1991; Newmark and Boswell 1994; Newmark et al. 1997; Micklem et al. 1997; van Eeden et al. 2001; Palacios et al. 2004). Depletion of EJC results in the skipping of several exons of *mapk* pre-mRNA, indicating the bias targeting of long intron genes (Ashton-Beaucage et al. 2010; Roignant and Treisman 2010). The GAAGA motif is a potential binding site for human EJC core eIF4AIII for gene expression regulation (Saulière et al. 2012). Mice MAGO plays a key function in brain size development by positively affecting microcephaly-associated *lissencephaly-1* (*LIS1*) expression during neurogenesis (Silver et al. 2010). Y14 and MAG-1 control germ line sex determination by inhibiting the proper expression of the transformer-2 (TRA-2) protein in *Caenorhabditis elegans* (Shiimori et al. 2013). A few plant studies have shown that EJC primarily targets the splicing of the *UDT1* transcript in rice and *Physalis* (Gong and He 2014; Huang et al. 2016). We found that plant EJC can affect transcription of genes associated with several specific pathways to produce related phenotypic variation in *Physalis*. The EJC homologues are closely interconnected at different levels, such as protein subcellular localization and protein dimerizations, to perform EJC functions in various organisms (Ashton-Beaucage et al. 2010; Roignant and Treisman 2010; Gong and He 2014; Choudhury et al. 2016). They can be interconnected at different steps of their own gene expression mechanisms in *Arabidopsis* (Mufarrege et al. 2011). This was not observed in rice or *Physalis* (Gong and He 2014; Huang et al. 2016). The differential extent of interconnection may contribute to the evolution of EJC diversity in function and mechanistic aspects. Examining more key species that reflect the phylogeny on the tree of life may contribute to understanding the evolutionary pattern of EJC targets and their related biological processes.

## Conclusions

The EJC is an important complex playing roles in RNA metabolism during eukaryotic life. EJC core genes share similarities in sequences and evolutionary history, but they play diverse developmental roles in different eukaryotic life forms. One such role is floral development in plants. In this study, we found that the EJC core in *Physalis* is primarily involved in pollen developmental pathways and associated with female functionality determination, Chinese lantern development and defense-related responses (Fig. 8). A primary target for *Physalis* EJC core is *PFDYT1*, which encodes orthologs of *UDT1*, the key bHLH transcription factor in pollen development in rice and other plants (Jung et al. 2005; Gong and He 2014; Jeong et al. 2014; Huang et al. 2016). This supports the essential and conserved role of plant EJC in pollen development. *Physalis* EJC also affected the gene expression in the developmental processes. The newly discovered roles of *Physalis* EJC core genes in defense-related processes was linked to *WRKY* genes, while the roles in Chinese lantern morphology (*PFeIF4AIII)* and carpel functionality (*PFY14*) appeared to be gene-specific. This finding needs further investigation. Our results suggest that EJC is involved in gene expression regulation and mRNA metabolism of multiple developmental processes in *Physalis*, and provide novel insights into the understanding of the functional evolution of EJC core genes. Further studies will help us understand the basis for the diversity and specificity of EJC target genes and the biological processes that are involved in species such as *P. floridana*. The adaptive value of EJC for plants during their evolution could also be revealed by advanced studies.

## Electronic supplementary material

Below is the link to the electronic supplementary material.

Supplementary material 1 (PDF 1347 KB)
