# Supplementary material for: Exon junction complex (EJC) core genes play multiple developmental roles in Physalis floridana
Source: Plant Mol Biol. 2018 Nov 13;98(6):545–63. doi: 10.1007/s11103-018-0795-9 (PMC6280879; doi:10.1007/s11103-018-0795-9)
Supplement: Supplementary file 1 — Supplementary material 1 (PDF 1347 KB) [file 11103_2018_795_MOESM1_ESM.pdf]

Supplementary material

Exon Junction Complex (EJC) Core Genes Play Multiple Developmental Roles in *Physalis floridana*

Pichang Gong[1,#], Jing Li[1,2,#], Chaoying He[1,2,*]

[1] State Key Laboratory of Systematic and Evolutionary Botany, Institute of Botany, Chinese Academy of Sciences, Beijing 100093 China;

[2] University of Chinese Academy of Sciences, Beijing 100049 China.

# These authors contributed equally to this work.

* For correspondence: E-mail: chaoying@ibcas.ac.cn; Tel: +86-10-62836085

Supplementary material contains:

Supplementary Figures S1-S13

Supplementary Tables S1-S3

PFMAGO-VIGS probe; 391 bp

(Sequence identity between *PFMAGO1* and *PFMAGO2* was 89.0%)

```
AGTTCGGGCACGAGTTCTTAGAATTCGAGTTTCGTCCCGACGGCAAGCTCCGTTATGCCAACAACTCCAACTACAAAAACGATACCATGATTCGTAAAGA
AATTTGGGCATGAGTTCTTAGAGTTTGAATTTAGGCCTGATGGCAAGCTTCGTTATGCTAACAATTCCAACTATAAGAACGATACAATGATTCGCAAGGA

GGTTTTCCTTACCCCTGCCGTTCTCAAAGAATGTCGCCGTATTGTTGCCGATAGCGAGATTATGAAGGAAGATGACAACAATTGGCCTGAACCGGATAGA
AGTCTTCCTCACCCCTTCTGTTCTCAAAGAATGCCGCCGCATTGTTGCTGATAGCGAGATCATGAAGGAAGATGATAACAACTGGCCTGAACCAGATAGA

GTGGGGAGGCAAGAGCTTGAGATTGTCATGGGAAATGAGCACATATCATTTACCACATCCAAGATTGGTTCACTCATGGATGTGCAGACCAGCAAAGATC
GTGGGCAGACAGGAGCTTGAGATTGTGATGGGAAATGAACACATATCATTCACTACATCTAAGATTGGTTCACTAATGGATGTGCAGACCAGTAAAGATC

CTGAGGGACTTCGTATCTTCTATTATCTTGTTCAGGACCTCAAGTGTTTTGTGTTCTCTCTCATCTCTCTCCATTTCAAGATCAAACCCAT
CTGAGGGACTTCGTATCTTCTATTATCTTGTTCAGGATCTGAAGTGTTTTGTGTTCTCTCTCATCTCGCTCCATTTCAAGATCAAACCCAT
```

PFY14-VIGS probe; 400 bp

```
GACGAAGACGTTGAGGGTTCATCACCTAGGGCAGCTATACCTAAGCTGAAATCTGCCATTACTGGTGGCGCTTCTAAGAAAATCAAAGGCCGTGGTTTCC
GTGAAGAGACTGCTGAAGCTGAACGAAATGCTCGCCTTTCTGCTCCTTTTGACTCTCTCGACTCCCAAGGTGGCCCTGGCCCTGAACGATCCATTGAAGG
ATGGATTGTTTTAGTCACTGGAGTTAATGAGGAGGCTCAAGAAGATCATCTTCATAATGCATTCGGCGAGTTCGGAGAGATCAAGAATTTGCATTTGAAT
CTGGATCGTCGTTCTGGATATGTCAAGGGTTATGCACTTATTGAATATGAAAACTTTGAAGAAGCAGAGAGAGCTATCAGTGAAATGGATGGGAATGAGC
```

PFeIF4AⅢ-VIGS probe; 462 bp

```
AGGTTGTACTGATTTCTGCCACTCTTCCAAATGAAATTCTGGAGATAACAAGCAAATTTATGACAGATCCTGTTCGGATTCTTGTGAAACGTGATGAATT
GACTCTAGAGGGCATCAAACAGTTCTTTGTAGCGGTGGAGAGAGAAGAGTGGAAATTTGATACACTCTGTGATCTTTATGATACACTCACGATCACCCAG
GCTGTTATTTTCTGCAACACAAAAAGAAAGGTTGATTGGCTAACAAACAAGATGCGCGAGAATAATTTTACAGTCTCATCTATGCATGGTGATATGCCAC
AGAAGGAAAGAGATGCTATTATGGCTGAGTTCCGAGGTGGCACGACTCGTGTTCTAATCACAACAGATGTTTGGGCAAGGGGACTGGATGTTCAACAGGT
GTCTCTGGTGATTAATTATGACCTTCCAAACAACAGAGAGCTCTACATTCATCGCATTGGTC
```

PFBTZ-VIGS probe; 370 bp

```
GGAAGCATGGGCAGTGGTGATCAGAACTTTCCTGGGACACCAGCCTTTTTGCCAGTCATGCAATTTGGTGGCCAGCACCGTGGTGGCATTCCTGCTGTTG
GCATGGCTTTTCCGGGATATGTTGGTCAGCCACAACTTGGAATGGGGAATTCTGAGATGACATGGTTGCCAGTTTTAGCTGGGGCTGCTGGAGCATTAGG
TGCAACATACTGTTCACCATATATTGCCATGGATGGTGCTTATCATGCTCGCCCTTCTGGGCAAATATCTTCTCTCACTGCTGCTCCAAGCAAAGAGAAC
AATACCAGTAAACCAAATAATGAAGTCAAGCCTCAACAGAGACCAGAGCTTTCAAATGACGACTTAGGCC
```

Fig. S1 Sequences used to trigger gene silencing in VIGS.
From top to bottom, the trigger sequence of *PFMAGO-*, *PFY14-*, *PFeIF4AIII-* and *PFBTZ*, and each length is given.

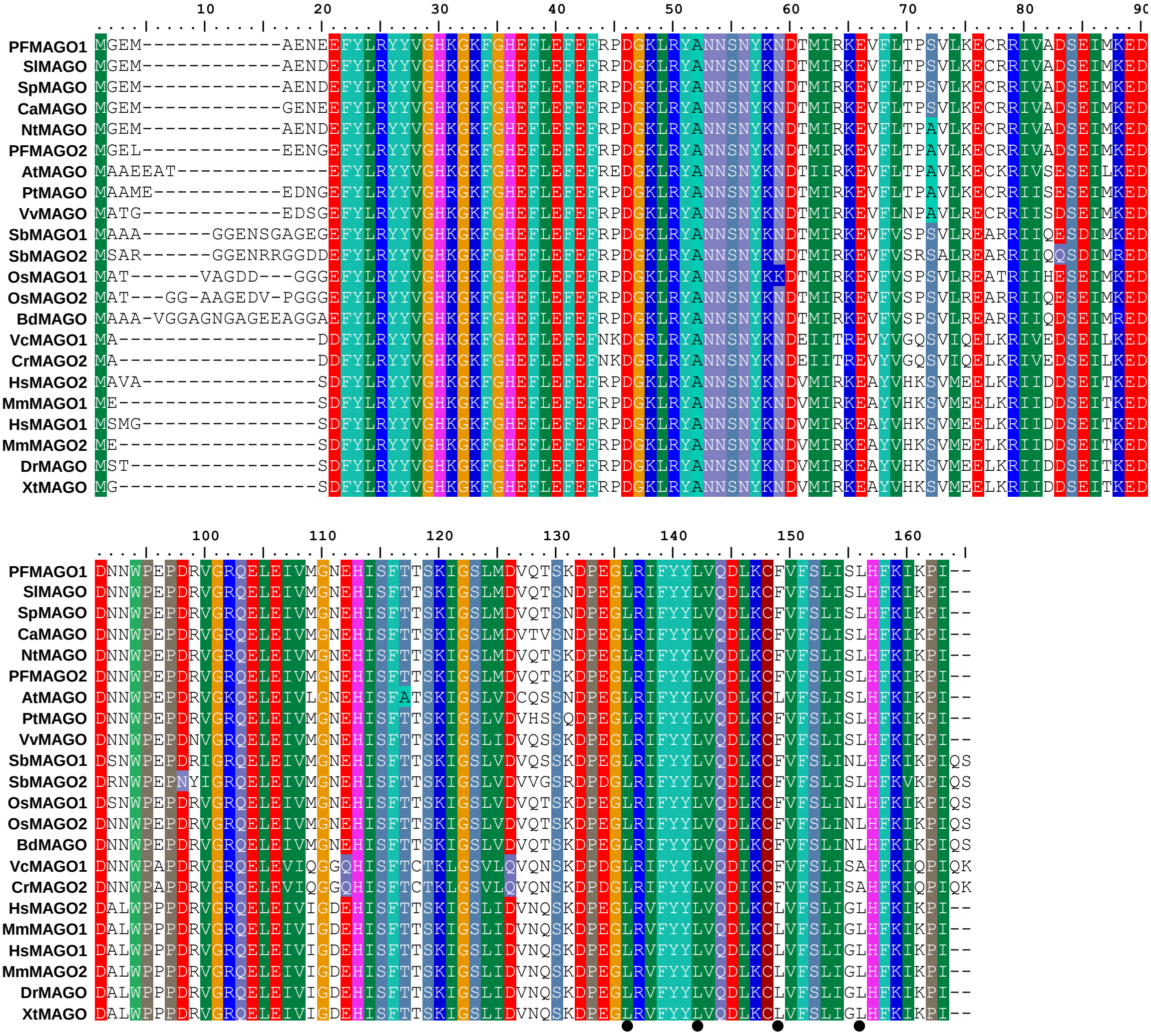

Fig. S2 Multiple sequence alignment of the MAGO protein family.

Shaded regions with color indicate 100% amino acid similarity. Four conserved leucine residues that constitute a potential leucine zipper in the C-terminus (Pozzoli et al. 2004; Chu et al. 2009) are labeled by black cycles.

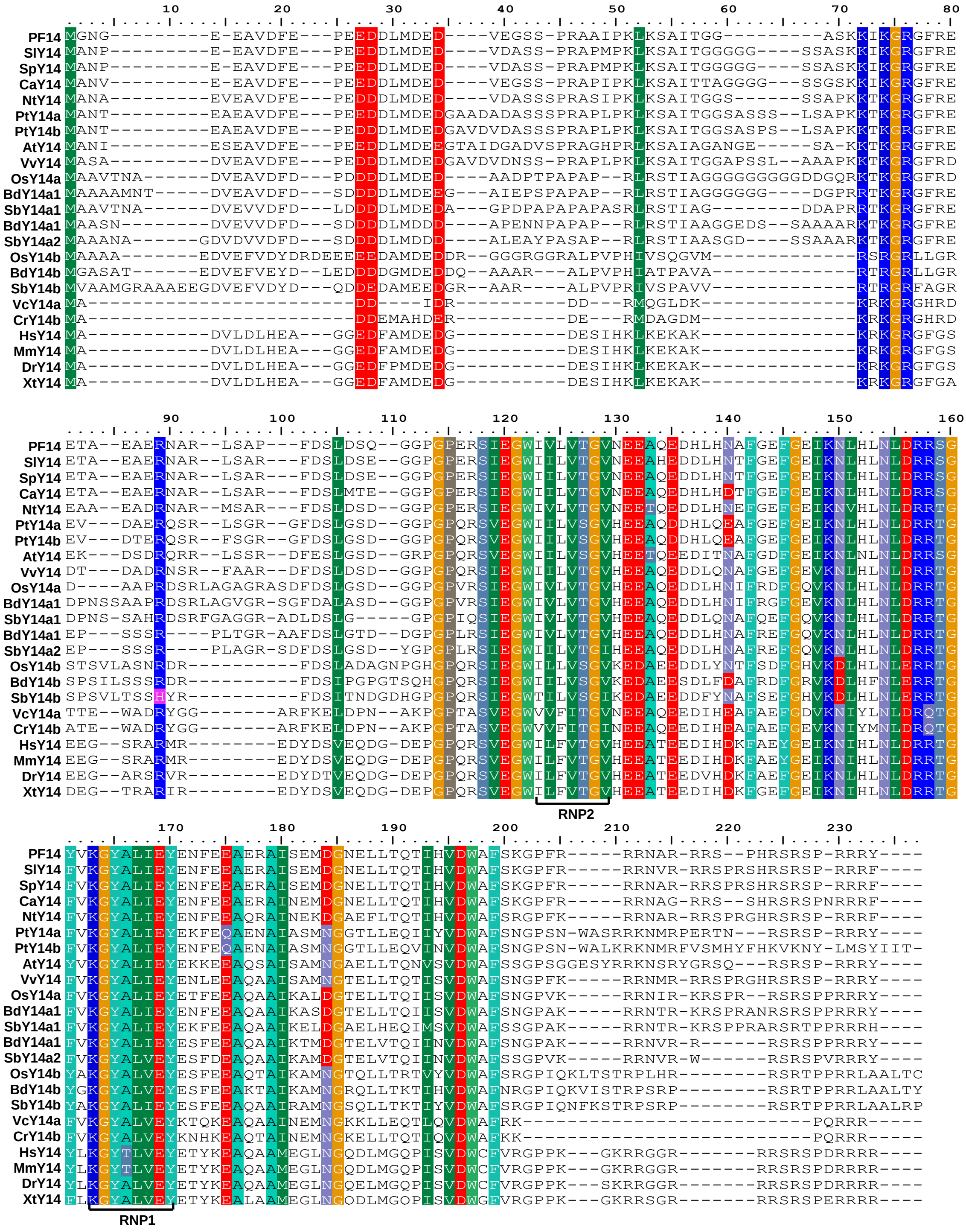

Fig. S3 Multiple sequence alignment of the Y14 protein family.
Shaded regions with color indicate 100% amino acid similarity. Conserved RNA recognition motifs of RNP1 and RNP2 (Lau et al. 2003; Shi and Xu 2003; Chu et al. 2009) are labeled in the middle area.

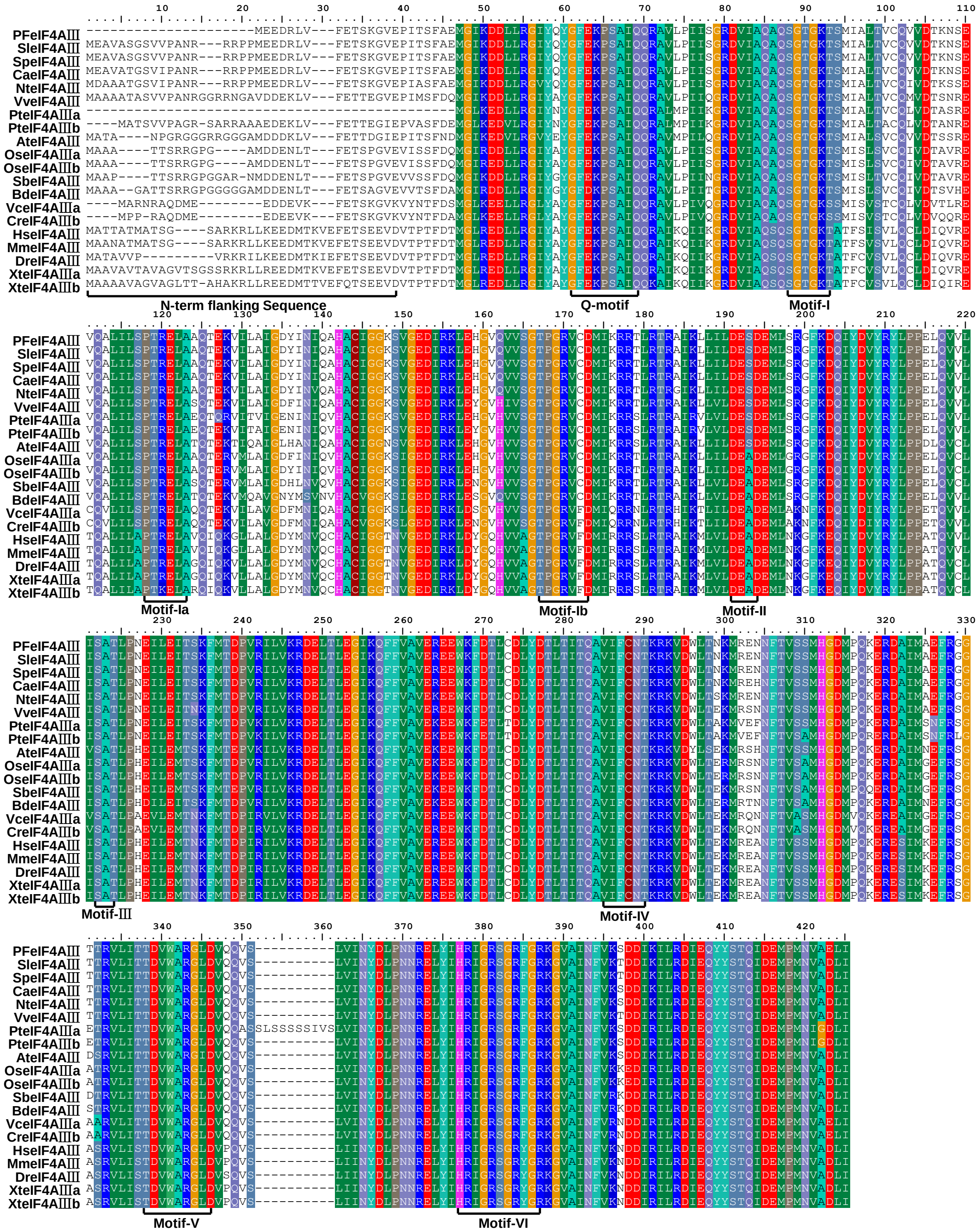

Fig. S4 Multiple sequence alignment of eIF4AIII protein family.
Shaded regions with color indicate 100% amino acid similarity. N-term flanking amino acid sequence and nine conserved motifs were labeled. Q-motif, motif-I, motif-Ia and motif-Ib were required for the ATP binding region; motif-II DEAD/DESD was involved in ATP hydrolysis and coupling ATP hydrolysis to helicase activity; motif-III SAT was critical for RNA unwinding; motif-IV and motif-V were equivalent to motif-Ia and motif-Ib and responsible to RNA binding; motif-VI was involved in the ATP hydrolysis-dependent RNA binding (Andersen et al. 2006; Cordin et al. 2006; Huang et al. 2016).

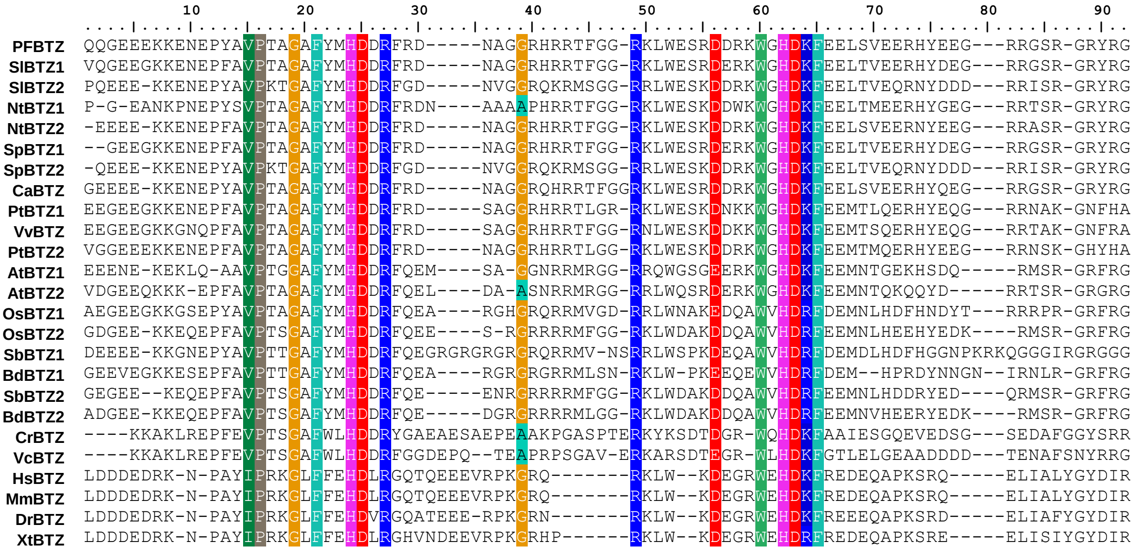

Fig. S5 Multiple sequence alignment of the SELOR domain in the BTZ protein family.
Only the SELOR domain (Degot et al. 2004), part of the whole MSA, is shown. Shaded regions with color indicate 100% amino acid similarity.

A

	PFMAGO1	PFMAGO2	PFY14	PFeIF4AIII	PFBTZ
Residue range	9-151	9-151	77-170	5-391	144-217
Tertiary structural modeling	Nt-β1-β2-β3-β4-α1-β5-β6-α2-α3-Ct	Nt-β1-β2-β3-β4-α1-β5-β6-α2-α3-Ct	Nt-β1-α1-β2-β3-α2-β4-β5-Ct	Nt-α1-α2-β1-α3-β2-α4-β3-α5-β4-β5-α6-α7-β6-α8-β7-β8-α9-β9-α10-β10-α11-β11-α12-β13-α13-β13-α14-α15-Ct	No α-helix nor β-sheet
Template (PDB ID)	X-Ray, 2.30 Å, PDB ID: 3EX7				
Identity (%)	82.52	81.25	54.29	78.97	27.63
GMQE	0.89	0.88	0.43	0.92	0.03
QMEAN4	-1.05	-0.95	-0.22	-0.46	-5.24
Organism	*Homo sapiens*				

B

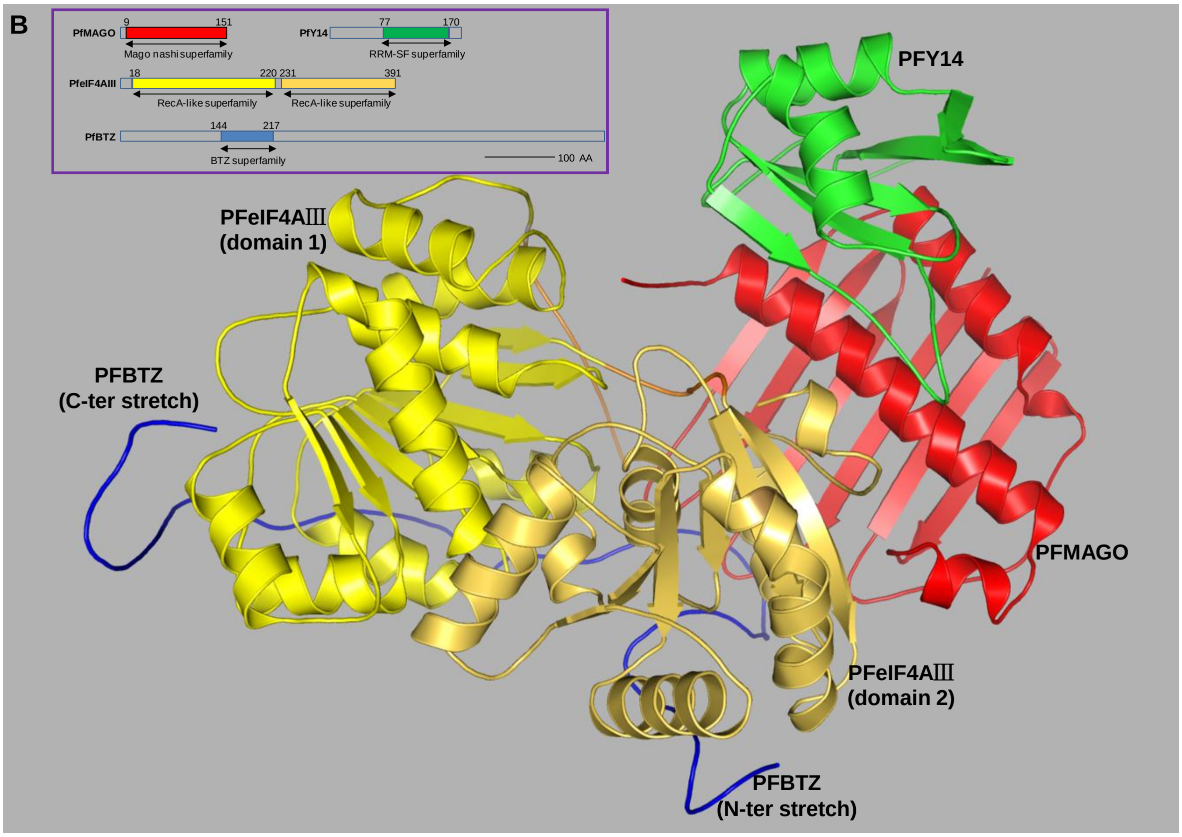

Fig. S6 Tetrameric structure modeling of EJC core components in *P. floridana*.
a Quality factors of *Physalis* EJC protein structure modeling. Nt, protein N-terminal; Ct, protein C-terminal; α, α-helix; β, β-sheet. **b** The tetrameric structure reconstruction of the *Physalis* EJC core based on human EJC (PDB ID: 3EX7). Conserved protein domains of EJC core components were predicted using the Pfam program and are presented in the pink box. The position of each conserved region or motif is given. Bar, 100 amino acids (AA).

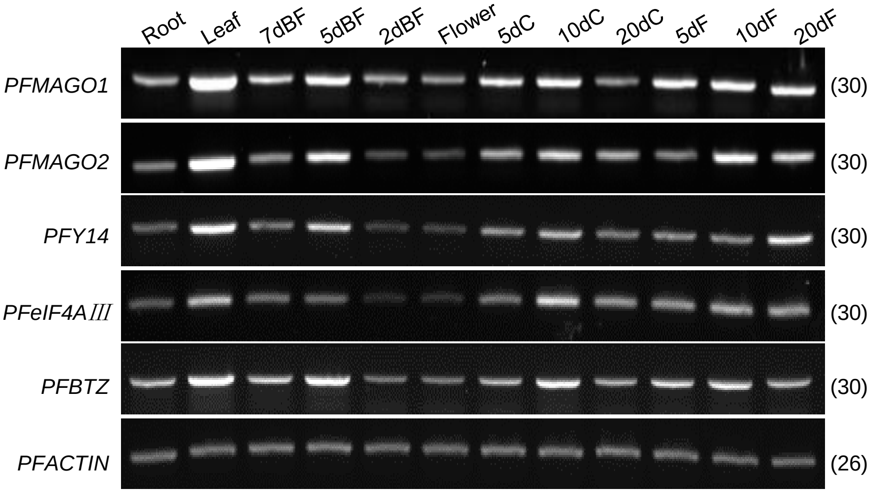

Fig. S7 Expression of *Physalis* EJC core genes by semi-quantitative RT-PCR. The total RNAs from roots, leaves, flower buds (7dBF, 5dBF, and 2dBF abbreviated for the flower buds of 7-, 5-, and 2-d before flowering), blooming flowers, fruiting calyx (5dC, 10dC and 20dC is short of the calyx of 5-, 10- and 20-d after fertilization) and developing berries (5dF, 10dF and 20dF represent fruits of 5-, 10- and 20-d after fertilization) were subjected to RT-PCR. The PCR products of 30 cycles for each EJC genes were demonstrated; 26 cycles of *PFACTIN* gene were shown as a loading control.

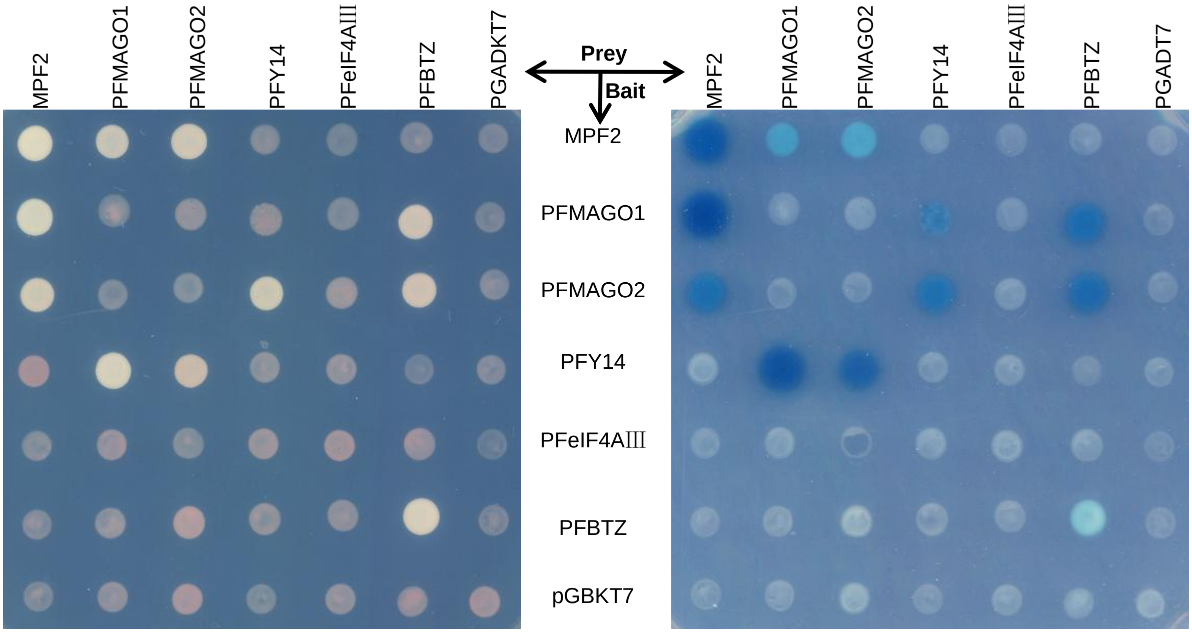

Fig. S8 PPIs among *Physalis* EJC core proteins revealed by yeast two-hybrid analysis.
Yeast cell growth on a high-stringency selective plate (SD/-Leu-Trp-His-Ade) (Left panel) and nonlethal β-galactosidase tests were used to confirm the interactions (right panel). The empty pGADT7 and pGBKT7 constructs were used as the negative controls.

A

Gene/VIGS	Infected seedling number	Mutated seedling number	Gene expression of mutants at 14 DAI compared to NC	Survival number (ratio)	Pollen viability	Fruit setting rate by self / cross-pollination
WT	-	-	1.00±0.05 (*PFMAGO1*)<br>1.06±0.16 (*PFMAGO2*)<br>0.99±0.21 (*PFY14*)<br>0.95±0.11 (*PFeIF4AⅢ*)<br>1.03±0.18 (*PFBTZ*)	30 (100%)	96.1%	95.5%/94.3%
TRV2 (NC)	30	-	1.0 (for each gene)	30 (100%)	95.8%	95.3%/94.6%
PFMAGO	120	115	0.26±0.13 (*PFMAGO1*)<br>0.32±0.12 (*PFMAGO2*)	25 (21.7%)	45.2%	35.3%/89.5%
PFY14	120	112	0.22±0.15	0 (0)	35.3%	0/0
PFeIF4AⅢ	120	113	0.35±0.21	44 (38.9%)	90.9%	95.2%/94.1%
PFBTZ	120	115	0.33±0.18	115 (100%)	50.1%	67.5%/92.3%

B

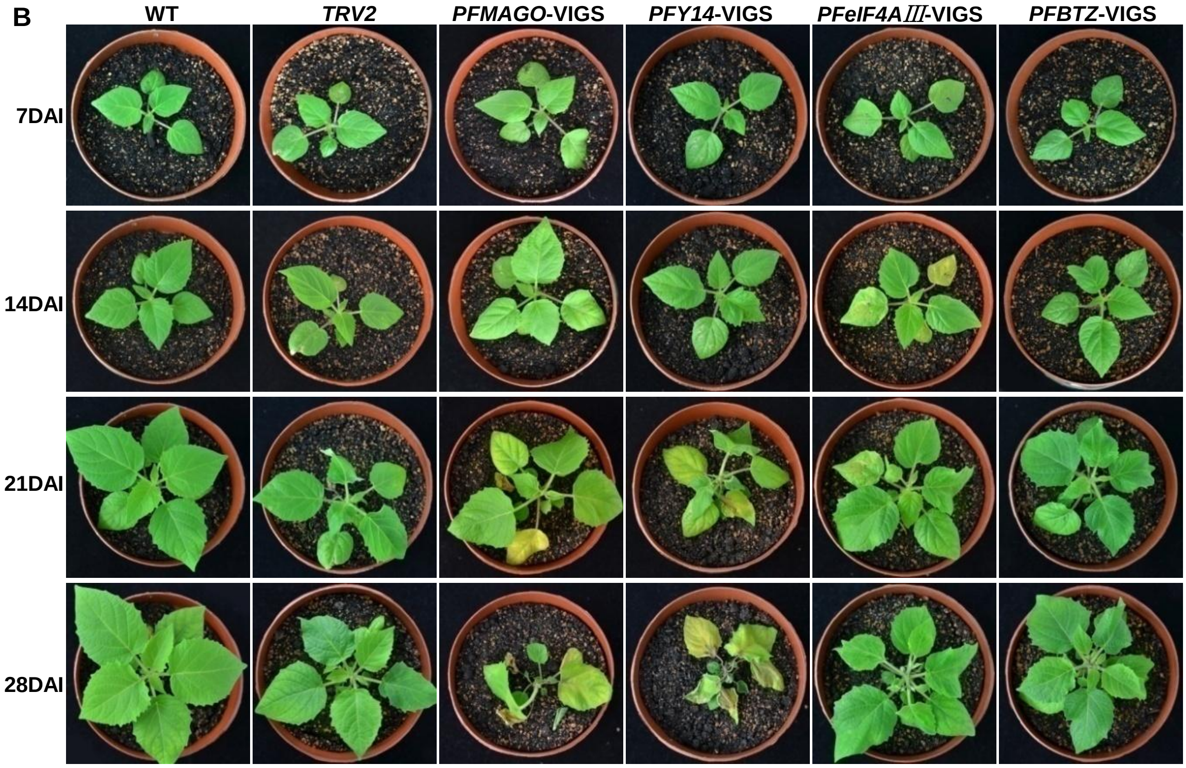

Fig. S9 Overall analysis of EJC core gene-VIGS in *P. floridana*.
a Evaluated parameters are as indicated. **b** Phenotypic variation of VIGS plants at vegetative stages compared to WT and NC. 7DAI, 14DAI, 21DAI and 24DAI indicated 7-, 14-, 21- and 24-d after infection, respectively.

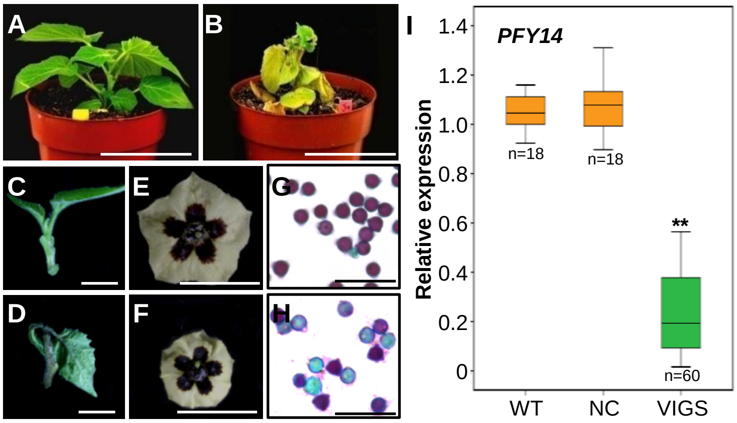

Fig. S10 *PFY14*-VIGS analysis in *P. floridana*.
a, b Plant growth of NC and *PFY14*-VIGS plants at 28DAI stage. **c, d** Shoot apex of NC and *PFY14*-VIGS plants. **e, f** Flower morphology of NC and *PFY14*-VIGS plants. **g, h** Pollen viability of NC and *PFY14*-VIGS plants, which was evaluated by I_2-KI staining. **i** The *PFY14* expression in flowers of plants as indicated. WT, wild type; NC, negative control; VIGS, *PFY14*-VIGS flowers showing severe pollen abortion. The flower number (n) investigated in each case was indicated. Bars, 10 cm in **a**, **b**, 1 cm in **c-f**, 100 μm in **g**, **h**. Significance relative to NC was evaluated by a two-tailed Student's *t* test, and ** indicates significance at $P < 0.01$.

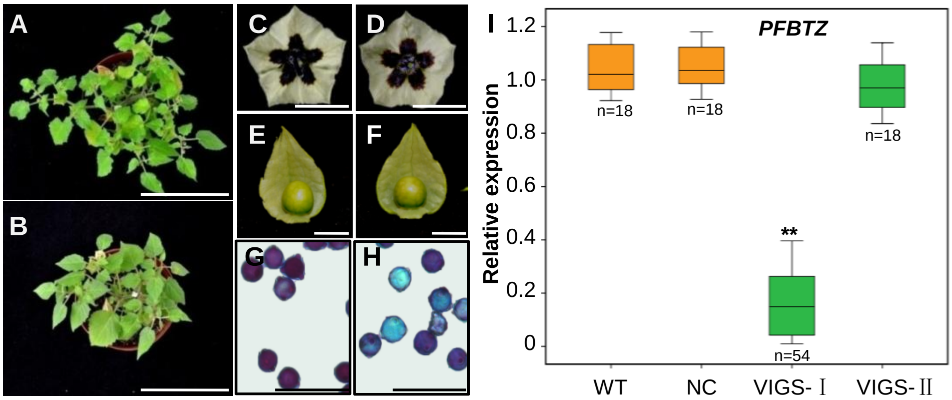

Fig. S11 *PFBTZ*-VIGS analysis in *P. floridana*.
a, b Phenotype of NC and *PFBTZ*-VIGS plants at the fruiting stage. **c, d** Flower morphology of NC and *PFBTZ*-VIGS plants. **e, f** Fruit morphology of NC and *PFBTZ*-VIGS plants. **g, h** Pollen viability of NC and *PFBTZ*-VIGS flowers, which was evaluated by I_2-KI staining. **i** The *PFBTZ* expression in flowers of plants as indicated. WT, wild type; NC, negative control; VIGS-I, *PFBTZ*-VIGS flowers showing pollen abortion; VIGS-II, *PFBTZ*-VIGS flowers having WT-like pollen development. The flower number (n) investigated in each case was indicated. Bars, 10 cm in **a**, **b**, 1 cm in **c-f**, 100 μm in **g**, **h**. Significance relative to NC was evaluated by a two-tailed Student's *t* test, and ** indicates significance at $P < 0.01$.

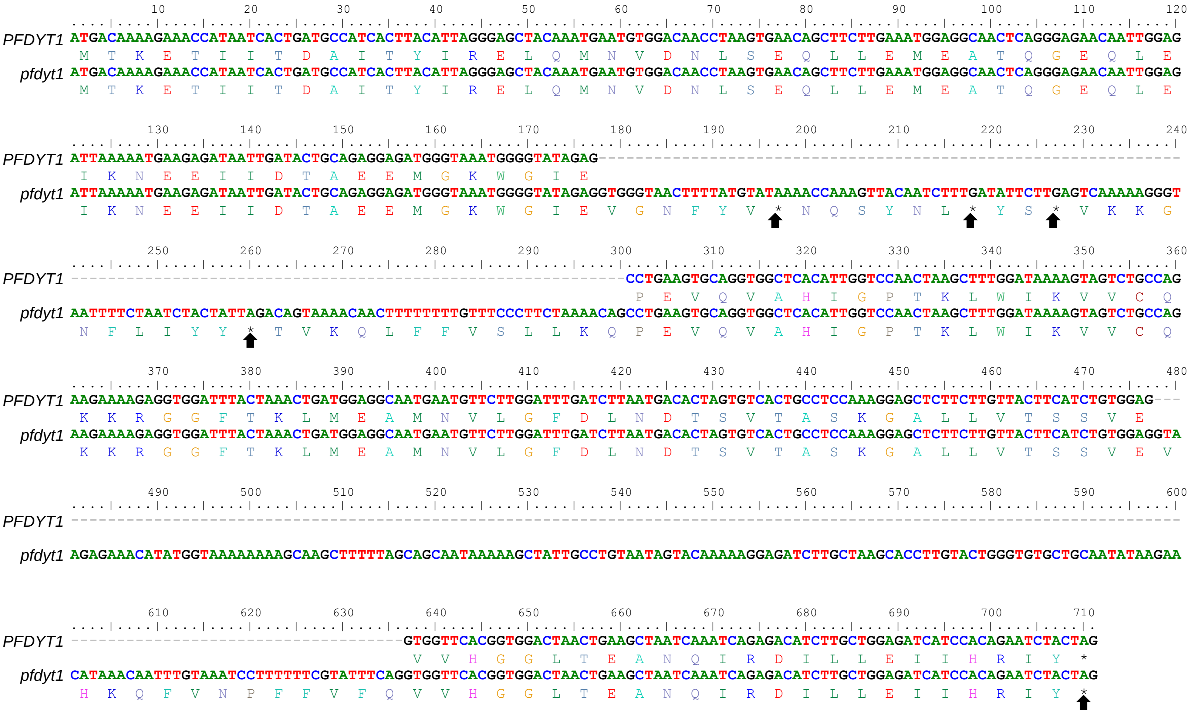

Fig. S12 Sequence comparison of different *PFDYT1* transcripts.
The normal cDNA of *PFDYT1* was 429 bp long, and putatively encoded a peptide of 142 amino acids (aa). Aberrant transcript (*pfdyt1*) was 711 bp, and maximally encoded a putative 65-aa peptide. Stop codon (*) was highlighted by black arrows. The - indicates the nucleotide acid gap.

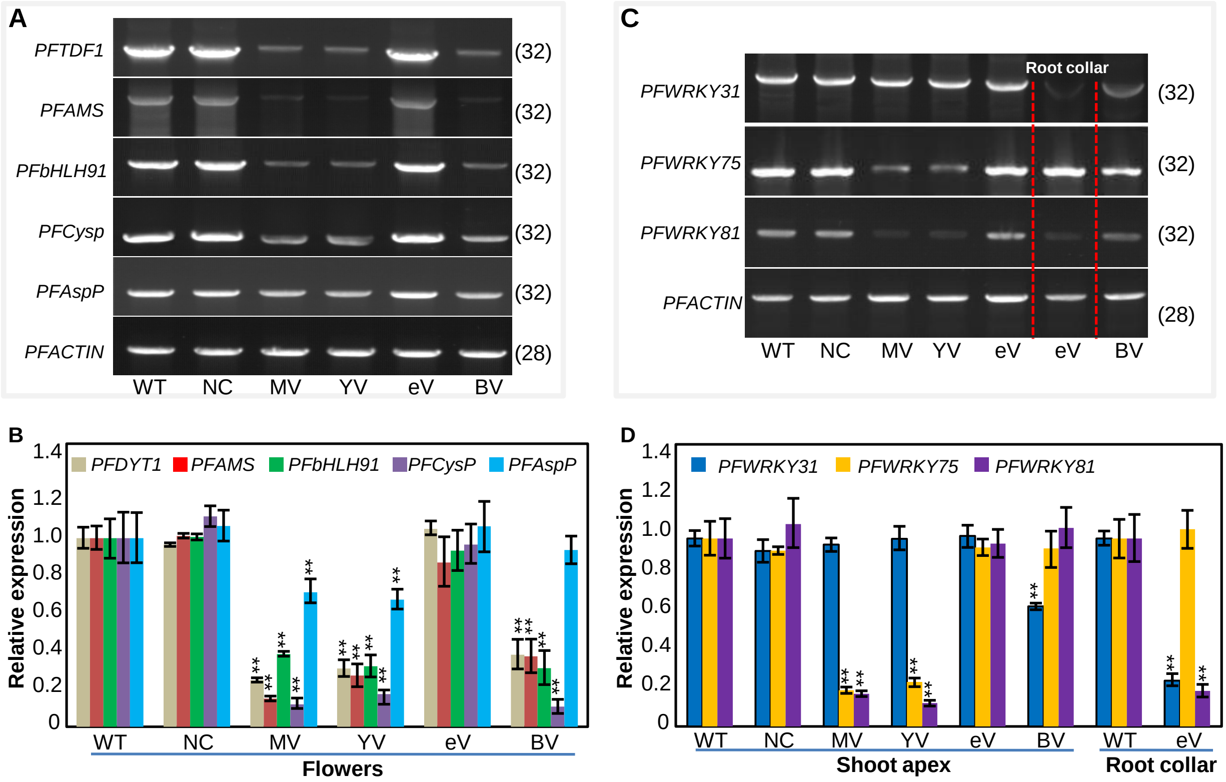

Fig. S13 Downregulations of EJC core genes affect gene expression.
a Full-length transcripts of the indicated genes in flower organs. WT, NC, MV, YV, eV and BV respectively represent wild type, negative control (*TRV2*), and *PFMAGO*-, *PFY14*-, *PFeIF4AIII*- and *PFBTZ*-VIGS *P. floridana* plants. The cycle number is given in the bracket. **b** The gene expression in 7DBF floral buds of the indicated plants. **c** Full-length transcripts of *PFWRKY* family genes in shoot apex or root collar. WT, NC, MV, YV, eV and BV respectively represent wild type, negative control (*TRV2*), and *PFMAGO*-, *PFY14*-, *PfeIF4AIII*- and *PFBTZ*-VIGS *P. floridana* plants. The sample between the two dashed lines is root collar, and others are the shoot apex. The cycle number is given in the bracket. **d** The gene expression of *PFWRKY* family in the shoot apex or root collar of the indicated plants. Significance relative to WT was evaluated by two-tailed Student's *t* test, and ** indicates significance at $P < 0.01$.

Table S1 Primers used in the present study.

Usage	Primer name	Forward sequence	Reverse sequence
	PFMAGO1	AGAATTCGAGTTTCGTCCCGAC	TGACAATCTCAAGCTCTTGCCTC
	PFMAGO2	AGTTTTACCTGAGATATTACGTG	AAGGGGTGAGGAAGACTTCCTTG
	PFY14	AATCAAAGGCCGTGGTTTCCG	AGCCTCCTCATTAACTCCAGTG
	PFeIF4AⅢ	TATTATGGCTGAGTTCCGAGGTG	CGACCAATGCGATGAATGTAG
	PFBTZ	GTGGTGGCATTCCTGCTGTTG	TTCTGACGCTGGCCTAAGTCG
	PFDYT1 (E1F-E3R)	ATGACAAAAGAAACCATAATCACTG	CTAGTAGATTCCGTGGATGATCTC
	PFDYT1 (E1F-I1R)		GTTGTTTTACTGTCTAATAG
	PFDYT1 (I1F-E2R)	AATCTTTGATATTCTTGAGTC	TCCAAGAACATTCATTGCCTC
	PFDYT1 (I2F-E3R)	GCTAAGCACCTTGTACTGGG	CTAGTAGATTCCGTGGATGATCTC
qRT-PCR	*PFDYT1* (E3F-E3R)	CCTGAAGTGCAGGTGGCTCAC	
	PFTDF1	ACTAGCTGGGAGCAACTAGCTGGG	CTTTGTTTGACCCATCTTGAGCACC
	PFAMS	GGATTGCTCGAGTTGTTCAGTACG	TCCCGAATTTGATCTTCCGTTATC
	PFbHLH	TACCCAAGTTTAGGTCTGCAGAAG	GAGGTATGGAGCTTCAGGCATTGG
	PFAspP	GCTACCCTAGGAGTTGGACAACC	GCAAACACTGAGTCACTAGGTCCG
	PFCysP	GTTGTGGAAGCTGTTGGGCGTTCTC	GCCACTTATTGTAACTGCATGCTG
	PFWRKY31	GTCAGATGATGGATACAATTGGAG	GATATCGATATCGCTTGTAGTTTG
	PFWRKY75	TCTTGGGGCTAATGGCAAGCATGG	GGAAGCATAGATTTGCATCTGACTC
	PFWRKY81	TAATTCGTGGTCGGGAGTTTACC	GTCTTGAATTTGTTGCACTTGTTTG
	PFACTIN	AACCGGAATGGTGAAGGCTGG	CCATATCATCCCAATTGCTGAC
	PFDYT1	ATGACAAAAGAAACCATAATCACTG	CTAGTAGATTCCGTGGATGATCTC
	PFTDF1	ATGGGGAGGCCTCCTTGTTGC	CTAGTAAAAGGGATCCTCAGAG
	PFAMS	ATGGAGAGGCTTAGGCCTATTATG	GTGATGGCGATGTCGGTGAAAGTG
	PFbHLH	AGCATGAAGGAGTAGCTGAAGATG	GAGCTTCTTGGCTATTGCACTTGC
RT-PCR	*PFAspP*	GAACTTCAGACCCCTGTCACG	ATCAGGACTAAATCCAACAAGG
	PFCysP	ATGAAGCCATCTTATATTACTC	TCAATAATCCTCCTTAAGAGG
	PFWRKY31	ATGGCTGCTTCAAGTTTCTCTTTTC	TCAGCAAAGAAATGACTCCATAAAC
	PFWRKY75	TATTCGCCAATATGTCCATCG	TTAGAAGGAAGCATAGATTTG
	PFWRKY81	ATGGGATCTTCATTGCCGGAA	GTCTTGAATTTGTTGCACTTGTTTG
	PFACTIN	ATGGCTGATGGTGAGGATATCC	GAAGGACCAGACTCATCATACTC
	PFMAGO1	GGCCATGGGGGAATTGGAAGAGAATG	ATGGATCCGGGTTTGATCTTGAAATGGAG
	PFMAGO2	GGCCATGGGGGAGATGGCAGAGAAC	ATGGATCCGGGTTTGATCTTGAAATGGAG
Y2H	*PFY14*	ATCCATGGGGAACGGGGAGGAGGCAG	ATGGATCCTTAGTATCTCCTCCTGGGACTTC
	PFeIF4AⅢ	TGCCATGGAAGAAGACCGGTTAGTTTTCG	AAGGATCCTATCAATTCAGCCACGTTCATTGG
	PFBTZ	TGACATATGGCGAGTGCTTGGAGAAGCAG	AAGGATCCTCACTGATCAAACTTCATCTCCG
	PFMAGO1	CACGGGGGACTCTAGAATGGGGGAATTGGAAGAGAATG	CGATGGATCCACTAGTAATGGGTTTGATCTTGAAATG
	PFMAGO2	CACGGGGGACTCTAGAATGGGGGAGATGGCAGAGAAC	CGATGGATCCACTAGTAATGGGTTTGATCTTGAAATG
BiFC	*PFY14*	CACGGGGGACTCTAGAATGGGGAACGGGGAGGAGGC	CGATGGATCCACTAGTGTATCTCCTCCTGGGACTTC
	PFeIF4AⅢ	CACGGGGGACTCTAGAATGGAAGAAGACCGGTTAGTTTTC	CGATGGATCCACTAGTTATCAATTCAGCCACGTTCATTGG
	PFBTZ	CACGGGGGACTCTAGAATGGCGAGTGCTGGAGAAGCAG	CGATGGATCCACTAGTCTGATCAAACTTCATCTCCGT
	PFMAGO1	AACCATGGGGGAATTGGAAGAGAATG	ATACTAGTAATGGGTTTGATCTTGAAATGG
	PFMAGO2	ATCCATGGGGGAGATGGCAGAGAAC	ATACTAGTAATGGGTTTGATCTTGAAATGG
Subcellular localization	*PFY14-1302*	AACCATGGGGAACGGGGAGGAGGCAG	CCACTAGTGTATCTGGTCCTGGGACTTC
	PFeIF4AⅢ	CACCATGGAAGAAGACCGGTTAGTTTTC	CCACTAGTTATCAATTCAGCCACGTTCATTGG
	PFBTZ-1300	AATCTAGATGGCGAGTGCTGGAGAAGCAG	AAGGTACCCTGATCAAACTTCATCTCCGTATATC
	PFMAGO	ACGGATCCAGTTCGGGCACGAGTTCT	TGCTCGAGATGGGTTTGATCTTGAA
	PFY14	ACGGATCCGACGAAGACGTTGAGGG	TGCTCGAGGCTCATTCCCATCCATT
VIGS	*PFeIF4AⅢ*	ACGGATCCAGGTTGTACTGATTT	TGCTCGAGCGACCAATGCGATGAA
	PFBTZ	ACGGATCCGGAAGCATGGGCAGT	TGCTCGAGGGCCTAAGTCGTCATTTG

Table S2 Molecular information of the isolated *Physalis* EJC core genes.

Gene	Accession number	ORF length (bp)	Protein length (Amino acid)	Grand average of hydropathicity
PFMAGO1	EF205415	456	151	-0.523
PFMAGO2	EF205416	456	151	-0.508
PFY14	KF051015	567	188	-0.973
PFeIF4AIII	MH319840	1176	391	-0.171
PFBTZ	MH319841	2067	688	-1.024

Table S3 Sequences used in the present study.

Species	Abbreviation	Accession number			
		MAGO	***Y14***	***eIF4A3***	***BTZ***
Physalis floridana	PF	EF205415<br>EF205416	KF051015	MH319840	MH319841
Solamun lycopersicum	Sl	NM_001291909	NM_00129108	XM_004250609	XP_010318560<br>XP_004252328
Solamun pennellii	Sp	XP_015067364	XP_01508009	XP_015058616	XP_015070832<br>XP_015060990
Nicotiana tomentosiformis	Nt	XP_009593407	XP_009586749	XP_009616974	XP_009586607<br>XP_009595299
Capsicum annuum	Ca	XP_016558805.1	XP_016574886	XP_016554349	PHT88780
Populus trichocarpa	Pt	XM_006375098	XM_002299753<br>XM_002314049	XM_002306354<br>XM_002310627	XM_006369105<br>XM_002303242
Arabidopsis thaliana	At	NM_100094	NM_104029	NM_112866	NM_202458<br>NM_101397
Vitis vinifera	Vv	XP_002281294	XP_002281228	XP_002275011	XP_010654695
Oryza sativa	Os	KF051011<br>KF051008	KF051016<br>KF051017	NM_001050208<br>NM_001057041	XP_015650091<br>XP_015640548
sorghum bicolor	Sb	02g027660<br>07g027660	XP_002439249<br>XP_002457845<br>XP_002466233	04g038330	XP_002455883<br>XP-021303601
Brachypodium distachyon	Bd	XP_003573269	XP_003568971<br>XP_003567720<br>XP_003559268	XP_003570724	XP_003569296<br>XP_003565997
Volvox carteri nagariensis	Vc	XM_001770356	XP_002953417	XP_002958883	XP_002951521
Chlamydomonas reinhardtii	Cr	XM_001763749	XP_001696992	XP_001699375	XP_001698374
Homo sapiens	Hs	NM_002370<br>NM_018048	NM_005105	NM_014740	XM_005257163
Mus musculus	Mm	NM_001282737<br>NM_025564	NM_001102407	NM_138669	NM_138660
Danio rerio	Dr	NP_001017700	NP_001013363	NP_957372	NM_205716
Xenopus tropicalis	Xt	XP_002931471	NP_001039147	NP_001084200<br>NP_001107349	NM_001012652
